# Supplementary material for: Self-Healing Thiolated Pillar[5]arene Films Containing Moxifloxacin Suppress the Development of Bacterial Biofilms
Source: Nanomaterials (Basel). 2022 May 9;12(9):1604. doi: 10.3390/nano12091604 (PMC9102331; doi:10.3390/nano12091604)

Supplementary Materials

Self-healing Thiolated Pillar[5]arene Films Containing Moxifloxacin Suppress the Development of Bacterial Biofilms

Dmitriy N. Shurpik [1,*], Yulia I. Aleksandrova [1], Olga A. Mostovaya [1], Viktoriya A. Nazmutdinova [1], Regina E. Tazieva [1], Fadis F. Murzakhanov [2], Marat R. Gafurov [2], Pavel V. Zelenikhin [3], Evgenia V. Subakaeva [3], Evgenia A. Sokolova [3], Alexander V. Gerasimov [1], Vadim V. Gorodov [4], Daut R. Islamov [5], Peter J. Cragg [6] and Ivan I. Stoikov [1,*]

[1] A.M.Butlerov Chemical Institute, Kazan Federal University, Kremlevskaya, 18, 420008 Kazan, Russia; a.julia.1996@mail.ru (Y.I.A.); olga.mostovaya@mail.ru (O.A.M.); n-vika-art@mail.ru (V.A.N.);
reginatzv@gmail.com (R.E.T.); alexander.gerasimov@kpfu.ru (A.V.G.)
[2] Institute of Physics, Kazan Federal University, Kremlevskaya, 18, 420008 Kazan, Russia; murzakhanov.fadis@yandex.ru (F.F.M.); marat.gafurov@kpfu.ru (M.R.G.)
[3] Institute of Fundamental Medicine and Biology, Kazan Federal University, Kremlevskaya, 18,
420008 Kazan, Russia; pasha_mic@mail.ru (P.V.Z.); zs_zs97@mail.ru (E.V.S.); zhenya_mic@mail.ru (E.A.S.)
[4] Enikolopov Institute of Synthetic Polymeric Materials Russian Academy of Sciences (ISPM RAS),
Profsoyuznaya, 70, 117393 Moscow, Russia; gorodovvv@ispm.ru
[5] Laboratory for Structural Analysis of Biomacromolecules, Kazan Scientific Center of Russian Academy of Sciences, Lobachevskogo, 2/31, 420111 Kazan, Russia; daut1989@mail.ru
[6] School of Applied Sciences, University of Brighton, Huxley Building, Brighton, East Sussex BN2 4GJ, UK; p.j.cragg@brighton.ac.uk
* Correspondence: dnshurpik@mail.ru (D.N.S.); ivan.stoikov@mail.ru (I.I.S.)

1. Materials and methods **2**

2. NMR, MALDI TOF MS, IR spectra of the compounds 2, 3, 3/3S, 3/4S **3**

3. NMR study **10**

4. Dynamic light scattering **11**

5. UV-vis study **15**

6. Chromatographic study of 3/3S, 3/4S, (3S)n, (4S)n. **18**

8. Thermal Gravimetric Analysis of 3, 3n, 3/3S, 3/4S **19**

9. Morphology and Composition of 3/3Sn, 3/4Sn **21**

9.1. Transmission Electron Microscopy 21

9.2. Scanning Electron Microscopy 22

9.3 Atomic Force Microscopy 25

9.4 Optical Microscopy 25

10. EPR study **26**

Materials and methods

Table S1. Variation of the conditions for the oxidative oligomerization of the macrocycle **3**, **3S** and **4S**.

**№**	**mass ratio of reagents 3/3S**	**t, °C**	**solvent**	**cat**	**t, hour**	**Yield oligomerization product 3/3S, %**
1	1:1	25	THF	H_2O_2	24	15
2	1:3	25	THF	H_2O_2	24	53
**3**	**1:5**	**25**	**THF**	$\mathbf{H_2O_2}$	**24**	**70**
4	1:10	25	THF	H_2O_2	24	60
5	1:5	25	CH_3CN	I_2	7	15
6	1:5	25	-	H_2O_2	48	9
7	1:5	75	THF	I_2	36	24
8	1:5	0	DMF	I_2	10	-
9	1:10	25	-	I_2	26	-
10	1:5	25	THF	$FeCl_3$	42	31
**№**	**mass ratio of reagents 3/4S**	**t, °C**	**solvent**	**cat**	**t, hour**	**Yield oligomerization product 3/4S, %**
11	1:1	25	THF	H_2O_2	24	12
12	1:3	25	THF	H_2O_2	24	55
**13**	**1:5**	**25**	**THF**	$\mathbf{H_2O_2}$	**24**	**76**
14	1:10	25	THF	H_2O_2	24	59
15	1:5	25	CH_3CN	I_2	7	3
16	1:5	25	-	H_2O_2	48	6
17	1:5	75	THF	I_2	36	27
18	1:5	0	DMF	I_2	10	-
19	1:10	25	-	I_2	26	-
20	1:5	25	THF	$FeCl_3$	42	25

2. NMR, MALDI TOF MS, IR spectra of the compounds 2, 3, 3/3S, 3/4S

Figure S1. ^{1}H NMR spectrum of 4,8,14,18,23,26,28,31,32,35 – deca-[acylthioethoxy]-pillar[5]arene **(2)**. $CDCl_3$, 298 K, 400 MHz.

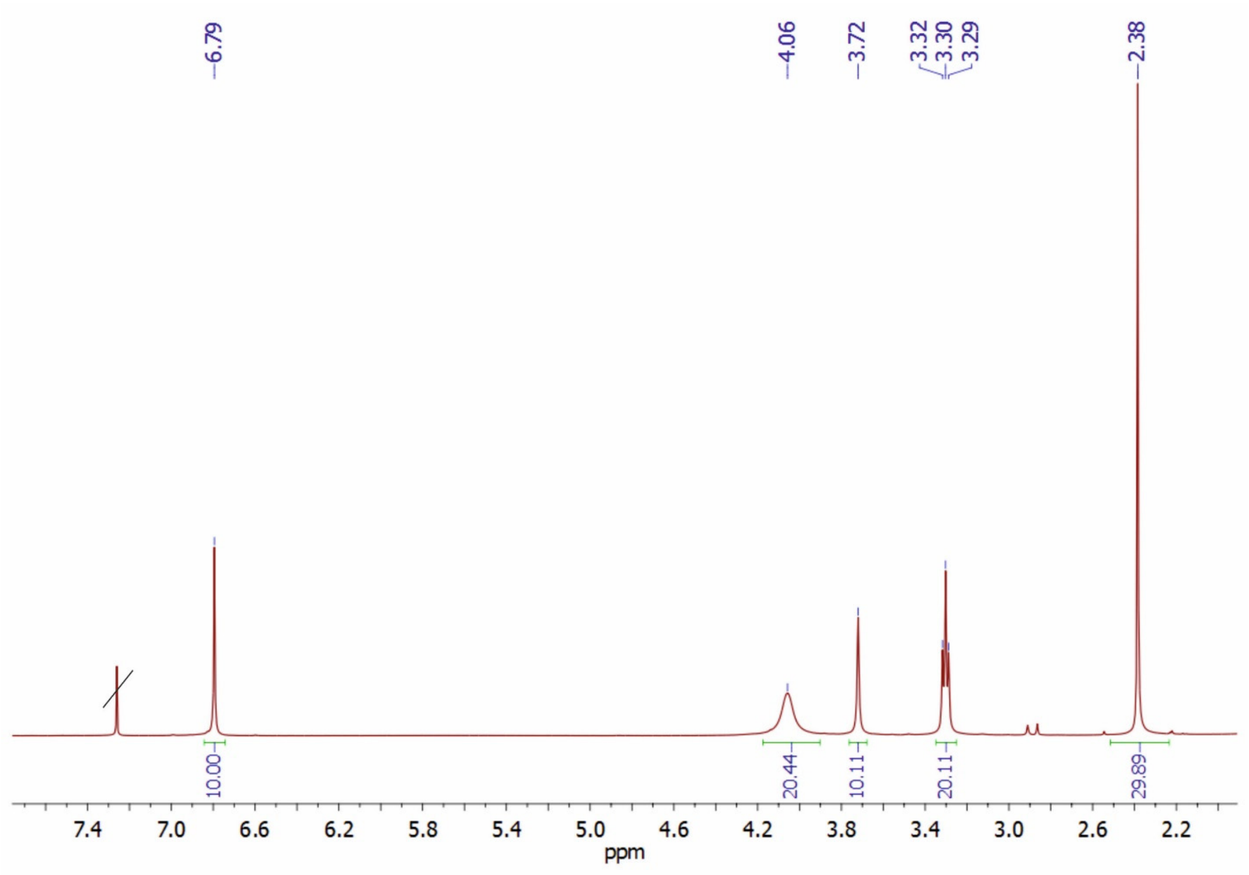

Figure S2. ^{1}H NMR spectrum of 4,8,14,18,23,26,28,31,32,35 – deca-[2-mercaptoethoxy]-pillar[5]arene **(3)**. $CDCl_3$, 298 K, 400 MHz.

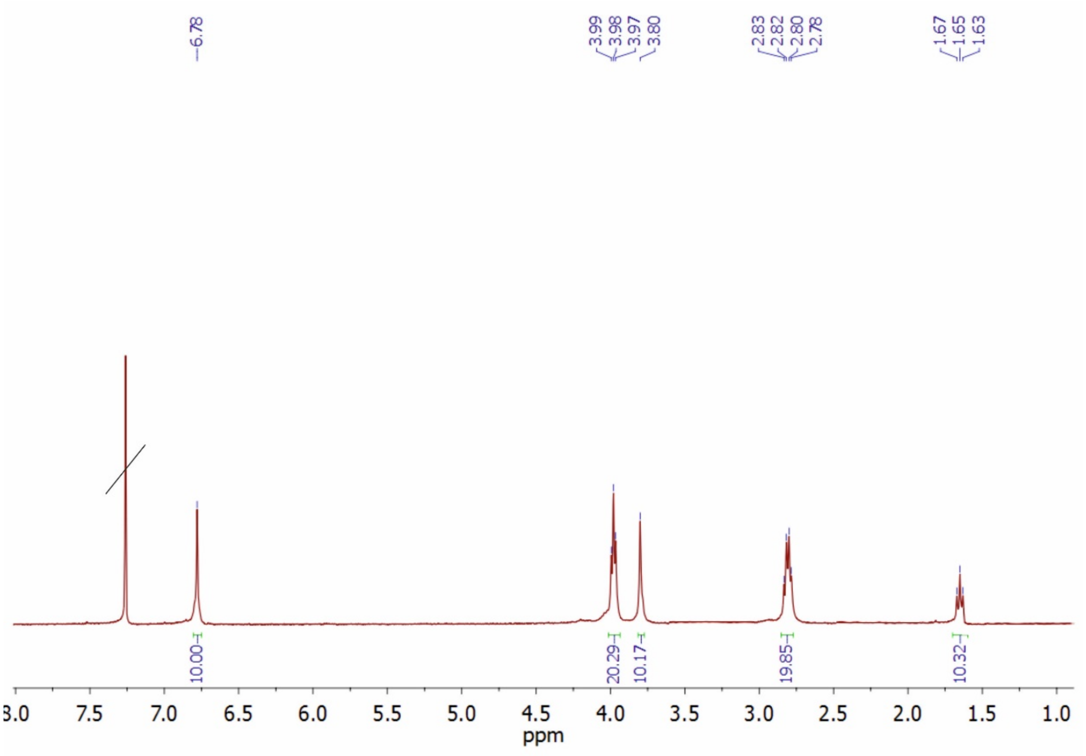

Figure S3. ^{13}C NMR spectrum of 4,8,14,18,23,26,28,31,32,35 – deca-[acylthioethoxy]-pillar[5]arene **(2)**. $CDCl_3$, 298 K, 400 MHz.

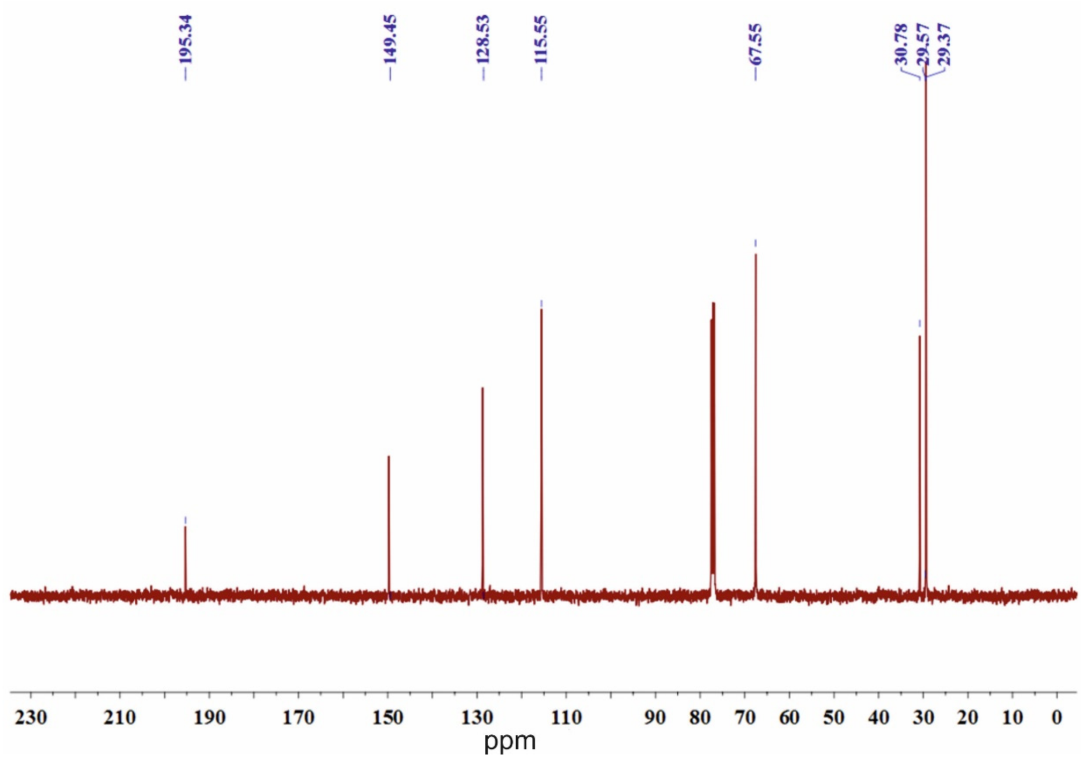

Figure S4. ^{13}C NMR spectrum of 4,8,14,18,23,26,28,31,32,35 – deca-[2-mercaptoethoxy]-pillar[5]arene **(3)**. $CDCl_3$, 298 K, 400 MHz.

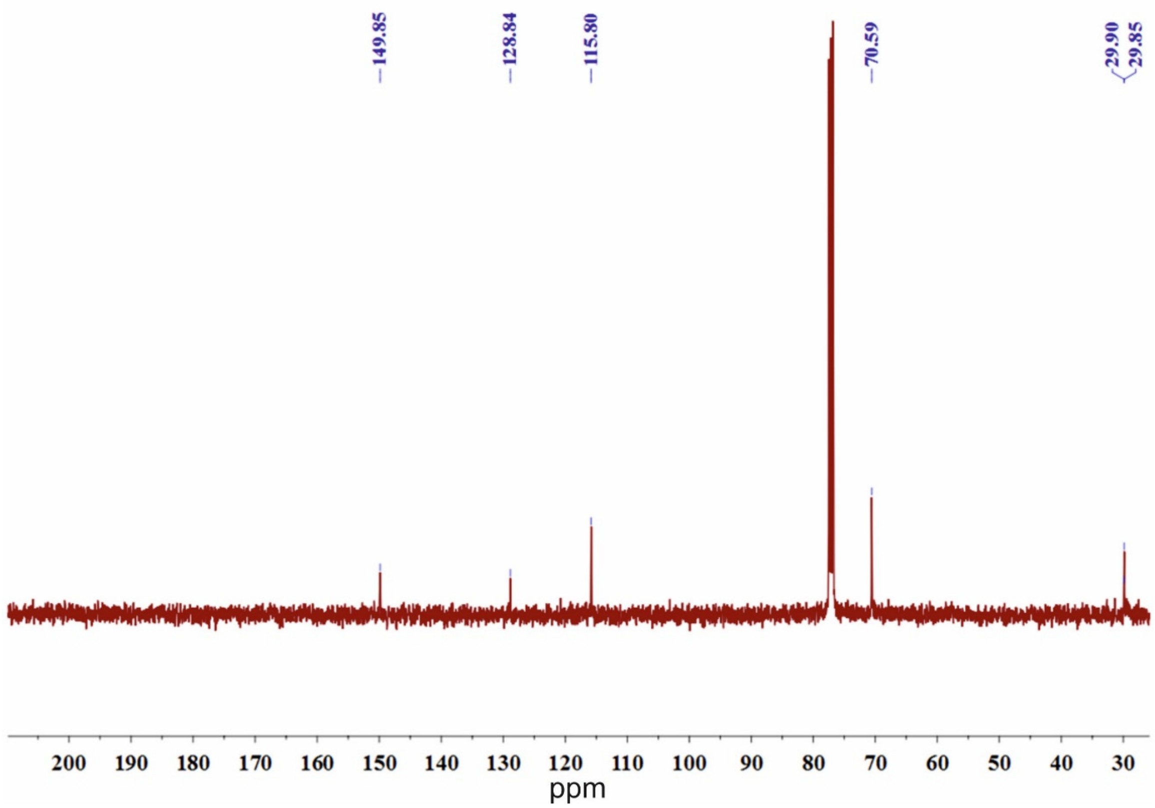

Figure S5. Mass spectrum (MALDI-TOF, 4-nitroaniline matrix) of 4,8,14,18,23,26,28,31,32,35 – deca-[acylthioethoxy]-pillar[5]arene **(2)**.

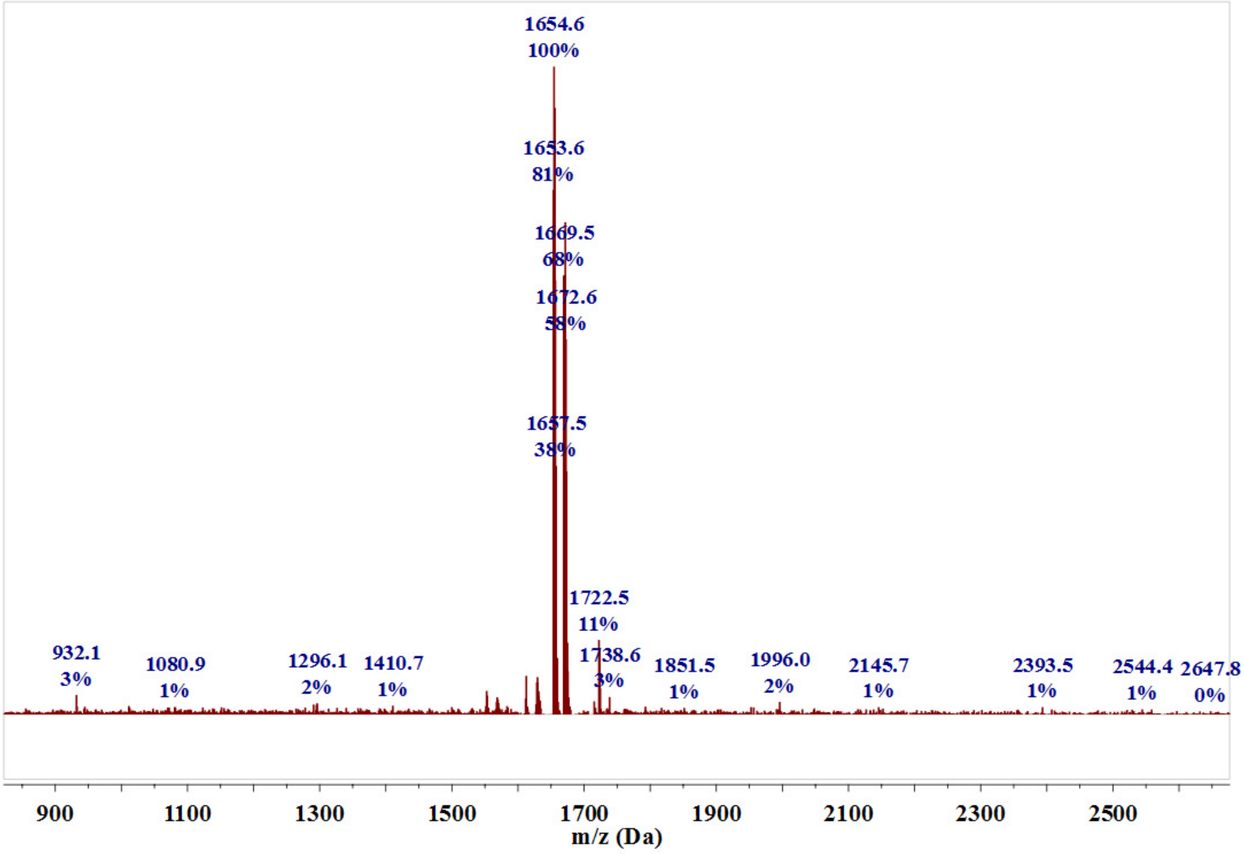

Figure S6. Mass spectrum (MALDI-TOF, 4-nitroaniline matrix) of 4,8,14,18,23,26,28,31,32,35 – deca-[2-mercaptoethoxy]-pillar[5]arene **(3)**

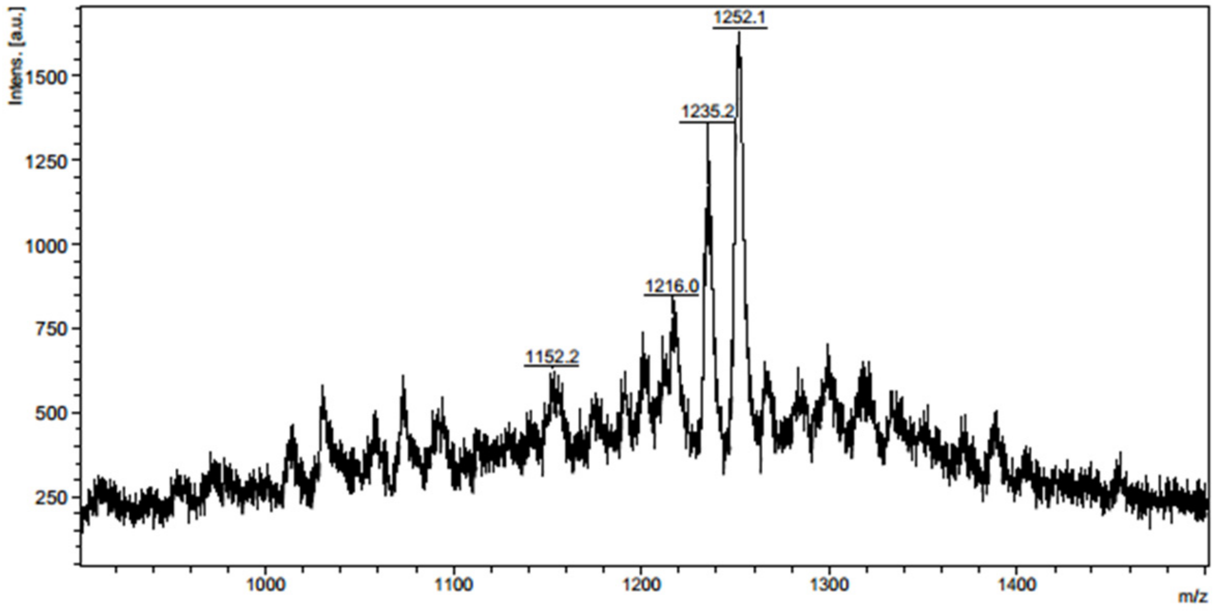

Figure S7. Mass spectrum (MALDI-TOF, 4-nitroaniline matrix) of tetrablock co-monomer, based on 4,8,14,18,23,26,28,31,32,35-deca-[2-mercaptoethoxy]-pillar[5]arene (**3**) and trimethylolpropane tris(3-mercaptopropionate), (**3/3S**).

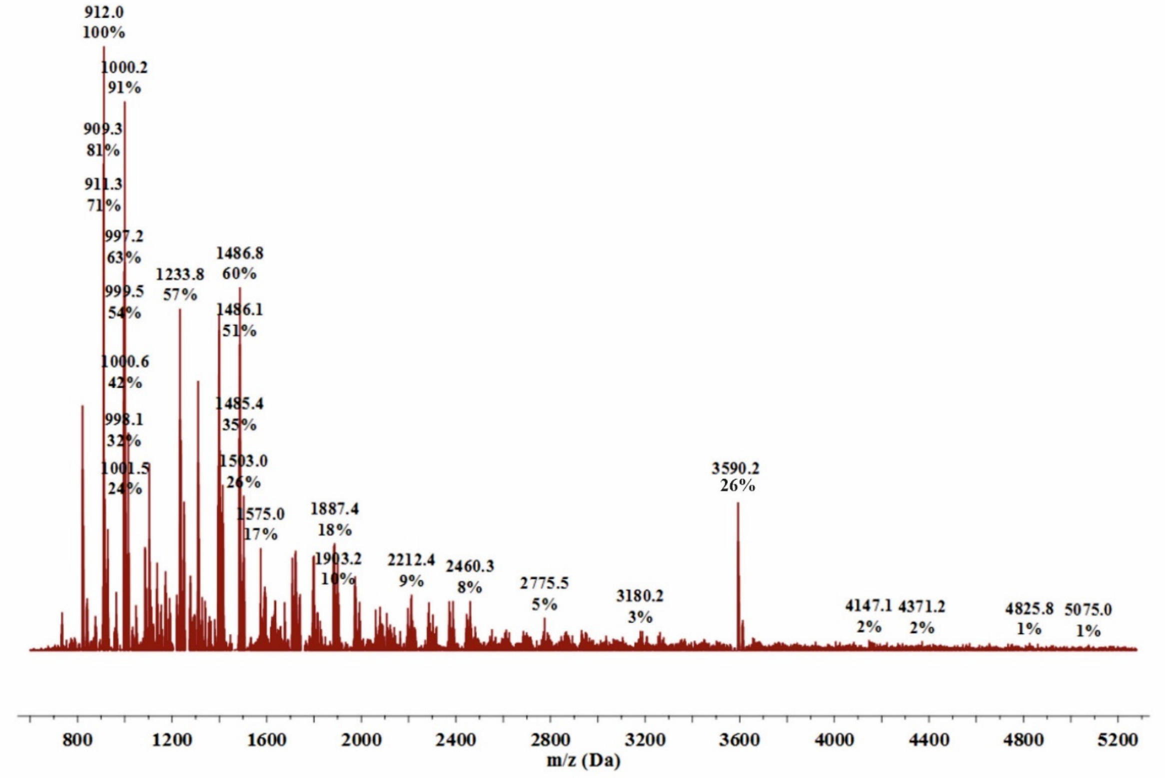

Figure S8. Mass spectrum (MALDI-TOF, 4-nitroaniline matrix) of tetrablock co-monomer, based on 4,8,14,18,23,26,28,31,32,35-deca-[2-mercaptoethoxy]-pillar[5]arene (**3**) and trimethylolpropane tris(3-mercaptopropionate), (**3/4S**).

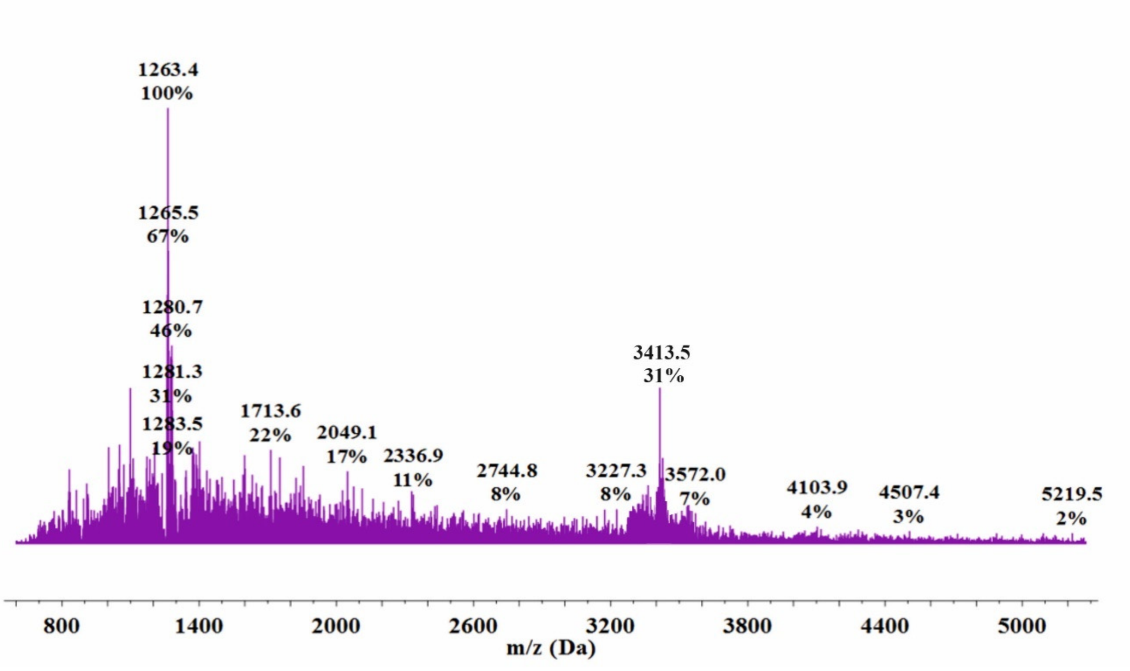

Figure S9. IR spectrum of 4,8,14,18,23,26,28,31,32,35 – deca-[acylthioethoxy]-pillar[5]arene **(2)**.

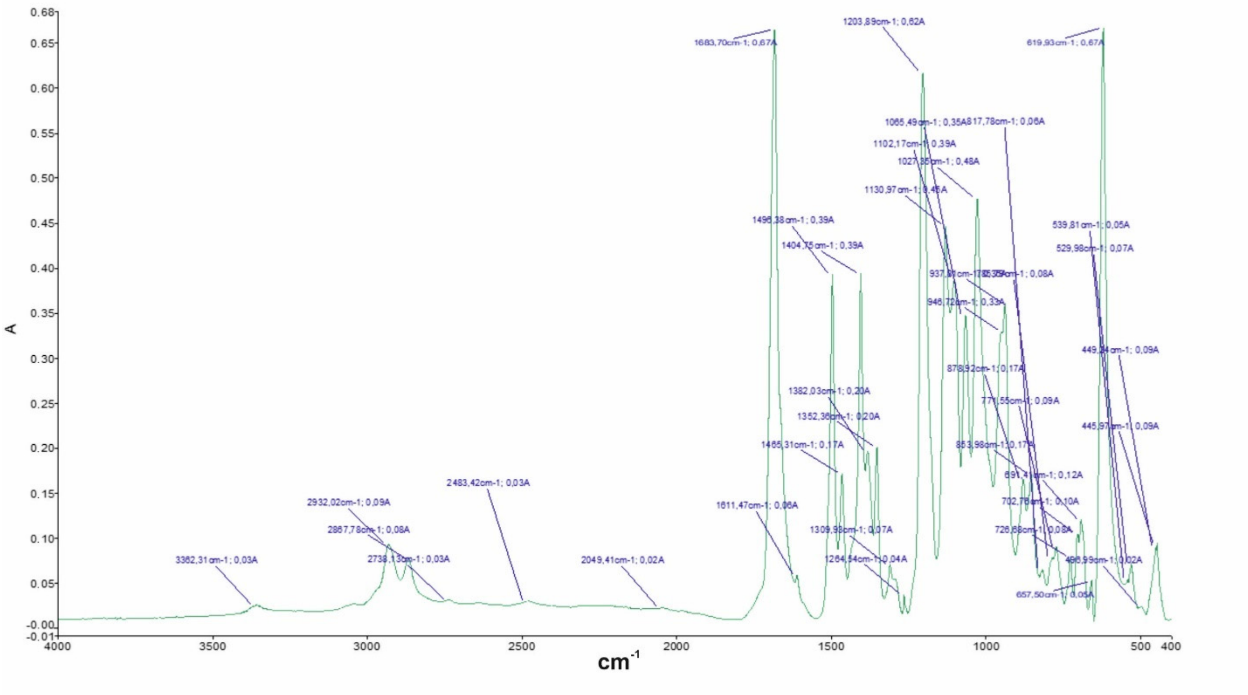

Figure S10. IR spectrum of 4,8,14,18,23,26,28,31,32,35 – deca-[2-mercaptoethoxy]-pillar[5]arene **(3).**

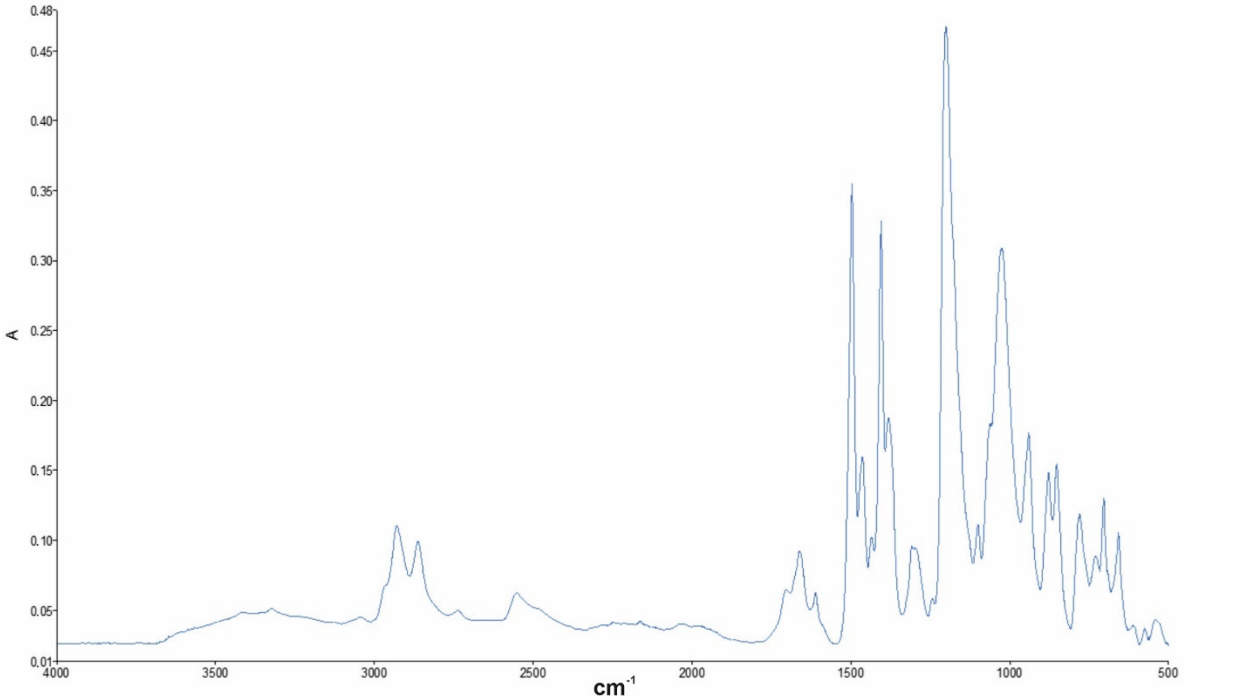

Figure S11. IR spectrum of 4,8,14,18,23,26,28,31,32,35-deca-[2-mercaptoethoxy]-pillar[5]arene **(3)** -based supramolecular polymer **(3)n.**

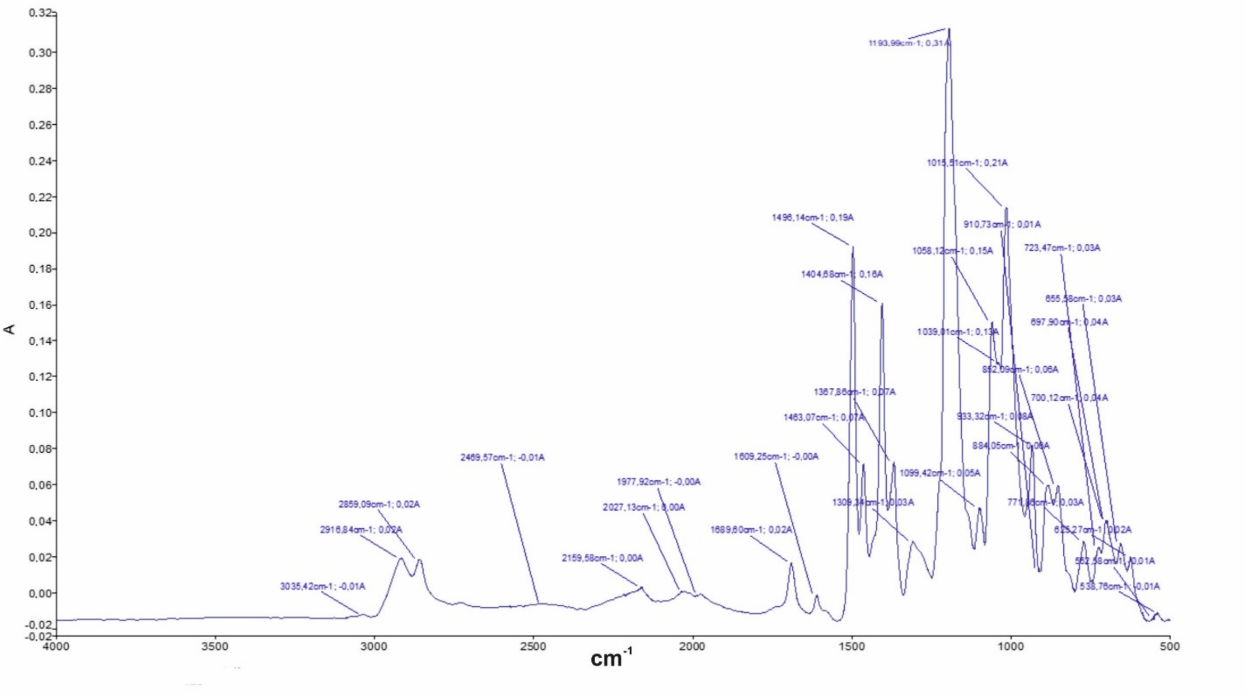

Figure S12. IR spectrum of tetrablock co-monomer, based on 4,8,14,18,23,26,28,31,32,35-deca-[2-mercaptoethoxy]-pillar[5]arene (**3**) and trimethylolpropane tris(3-mercaptopropionate), **(3/3S)**

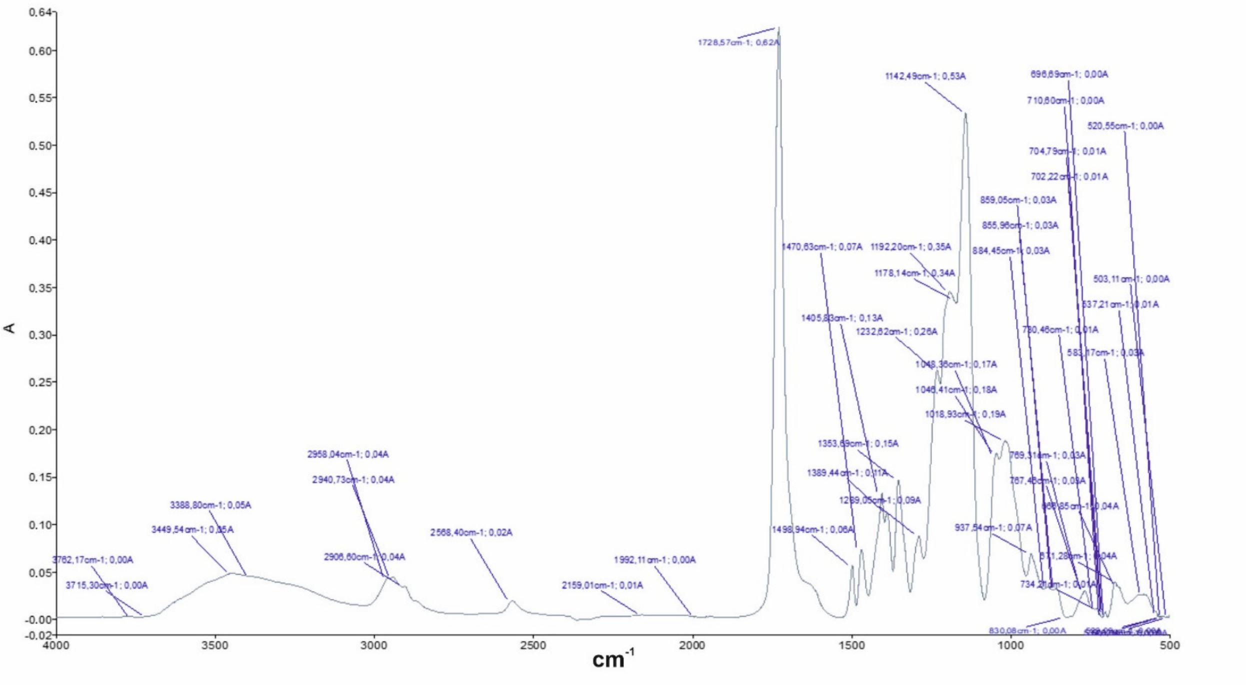

Figure S13. IR spectrum of tetrablock co-monomer, based on 4,8,14,18,23,26,28,31,32,35-deca-[2-mercaptoethoxy]-pillar[5]arene (**3**) and trimethylolpropane tris(3-mercaptopropionate), **(3/4S)**.

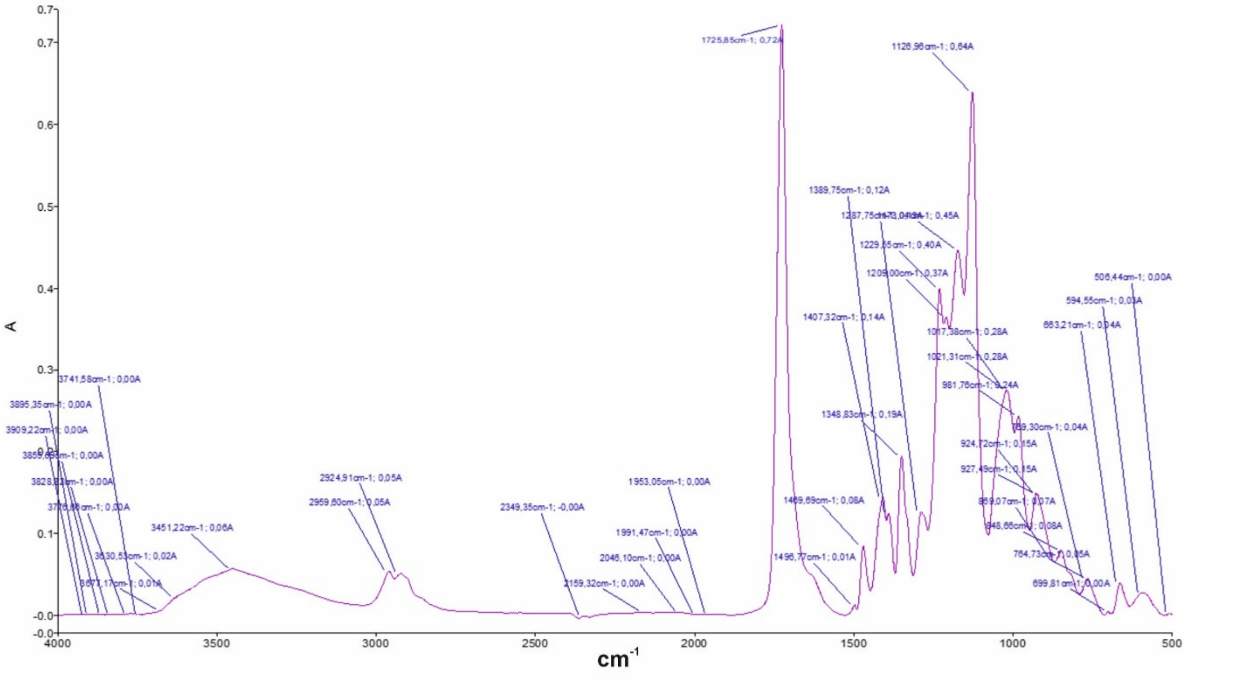

3. NMR study

Figure S14. (a) The 2D ^{1}H-^{1}H NOESY NMR spectrum of the **3** / moxi complex (2:1, 5×10^{-3} M) in $CHCl_3/CD_3OD$ = 100:1 at 25 °C; (b) 2D DOSY NMR **3** / moxi complex in $CHCl_3/CD_3OD$ = 100:1 at 25 °C (400 MHz, 298K).

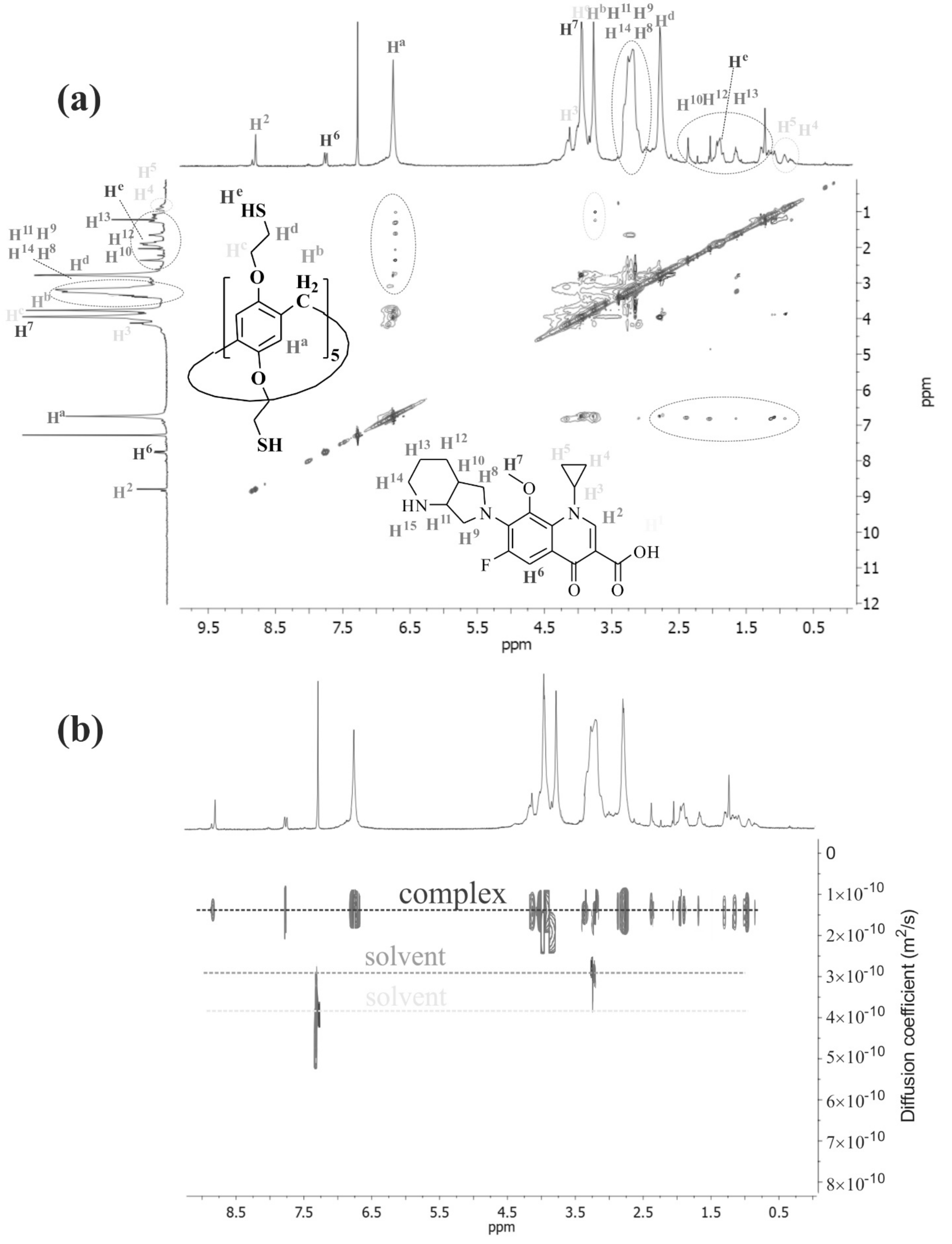

4. Dynamic light scattering

Figure S15. Size distribution of the particles by intensity for **3** (1×10^{-4} M) in solvent system THF: CH_3OH 100:1 (d=845 ± 303 nm, PDI= 0.42 ± 0.06)

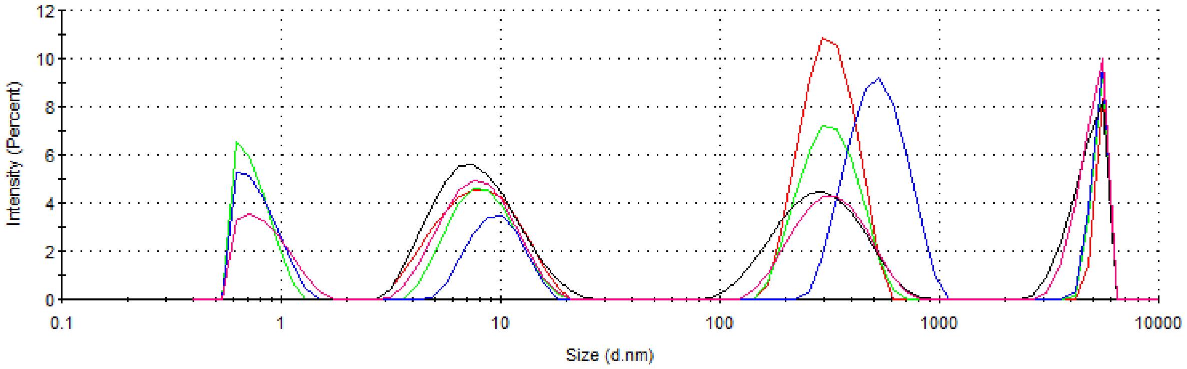

Figure S16. Size distribution of the particles by intensity for **3/3S** (1×10^{-5} M) in solvent system THF: CH_3OH 100:1 (d=782±254 nm, PDI= 0.650±0.174)

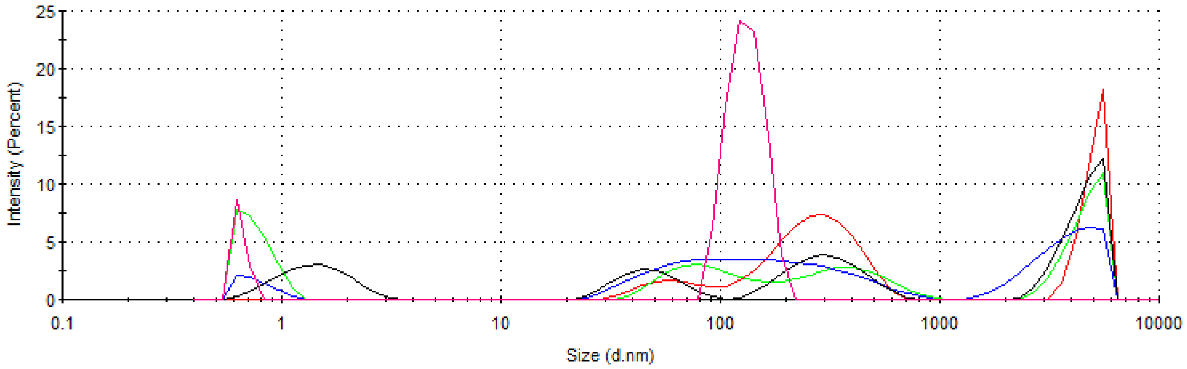

Figure S17. Size distribution of the particles by intensity for **3/4S** (1×10^{-5} M) in solvent system THF: CH_3OH 100:1 (d=640 ± 193 nm, PDI= 0.35 ± 0.02)

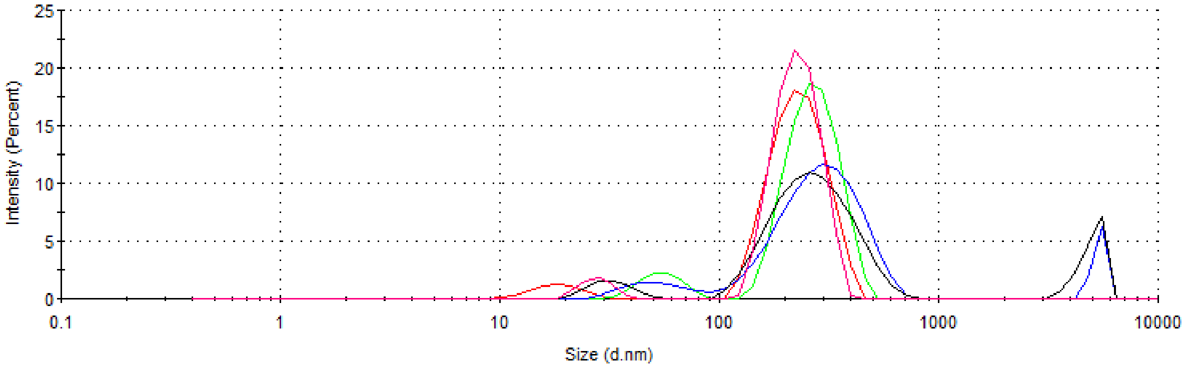

Figure S18. Size distribution of the particles by intensity for **moxi** ($1{\times}10^{-4}$ M) in solvent system THF: CH_3OH 100:1 (d=1269 ± 479 nm, PDI= 0.50 ± 0.14)

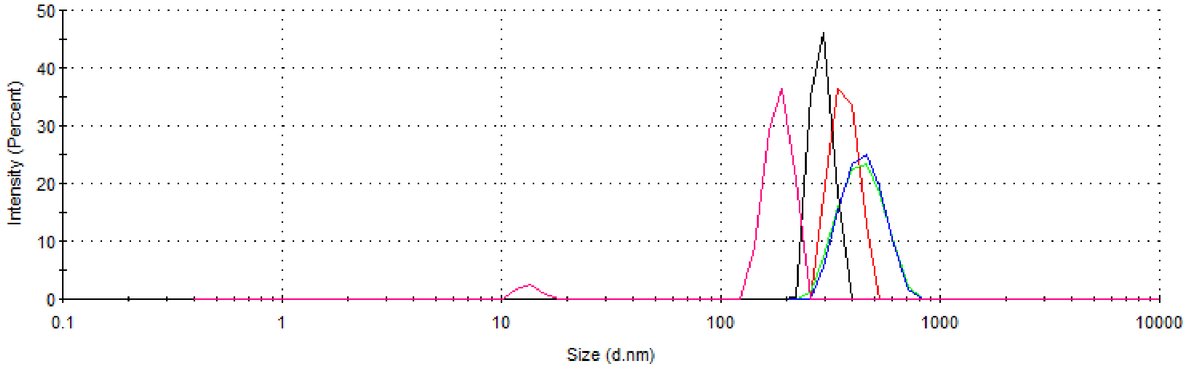

Figure S19. Size distribution of the particles by intensity for **BCl** ($1{\times}10^{-4}$ M) in solvent system THF: CH_3OH 100:1 (d=209 ± 21 nm, PDI= 0.24 ± 0.06)

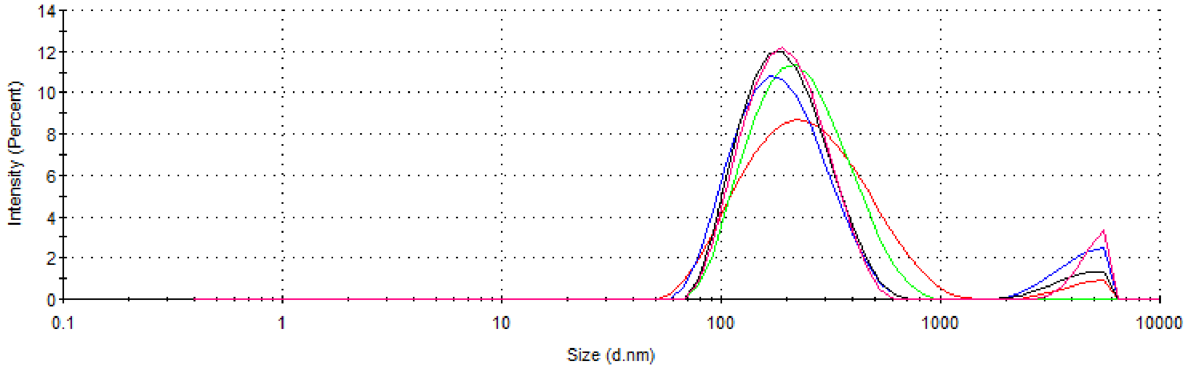

Figure S20. Size distribution of the particles by intensity for **3/3S (**$1{\times}10^{-5}$M) **+ moxi** ($1{\times}10^{-4}$ M) in solvent system THF: CH_3OH 100:1 (d=617 ± 203 nm, PDI= 0.34 ± 0.05)

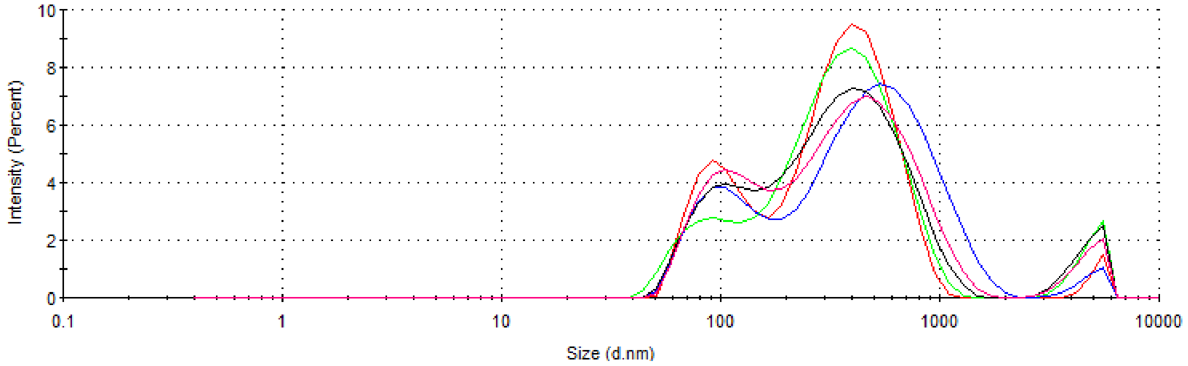

Figure S21. Size distribution of the particles by intensity for **3/4S (**$1{\times}10^{-5}$M) **+ moxi** ($1{\times}10^{-4}$ M) in solvent system THF: CH_3OH 100:1 (d=462 ± 13 nm, PDI= 0.28 ± 0.04)

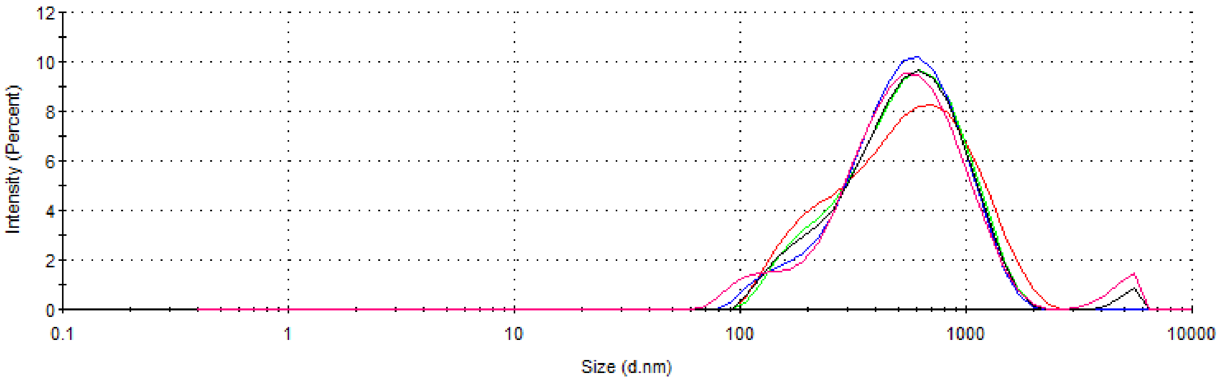

Figure S22. Size distribution of the particles by intensity for **(4S)n** (1×10^{-5}M) **+ moxi** (1×10^{-4} M) in solvent system THF: CH_3OH 100:1 (d=561 ± 7 nm, PDI= 0.24 ± 0.02)

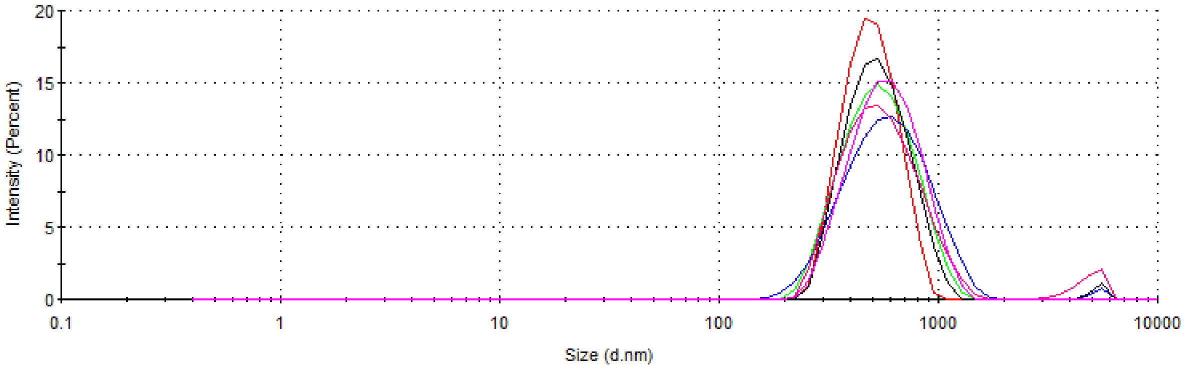

Figure S23. Size distribution of the particles by intensity for **3/3S** (1×10^{-5}M) **+ BCl** (1×10^{-4} M) in solvent system THF: CH_3OH 100:1 (d=556 ± 97 nm, PDI= 0.29 ± 0.05)

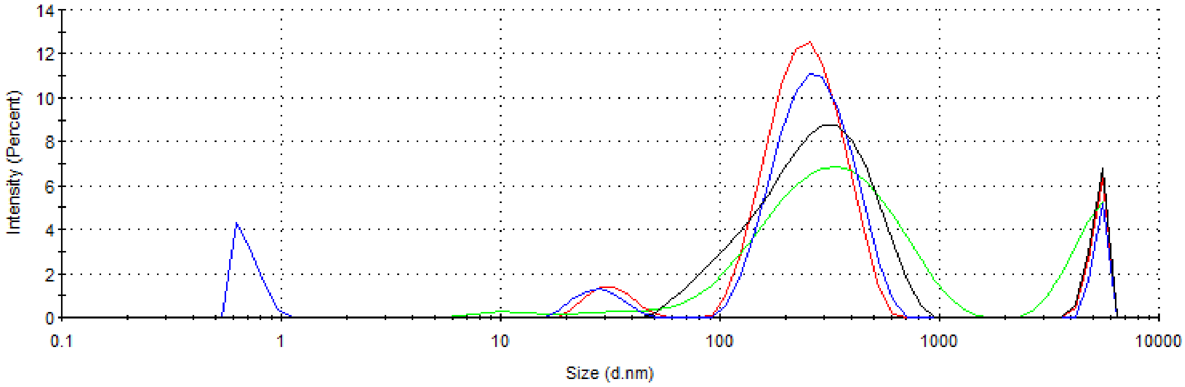

Figure S24. Size distribution of the particles by intensity for **3/4S** (1×10^{-5}M) **+ BCl** (1×10^{-4} M) in solvent system THF: CH_3OH 100:1 (d=553 ± 140 nm, PDI= 0.36 ± 0.10)

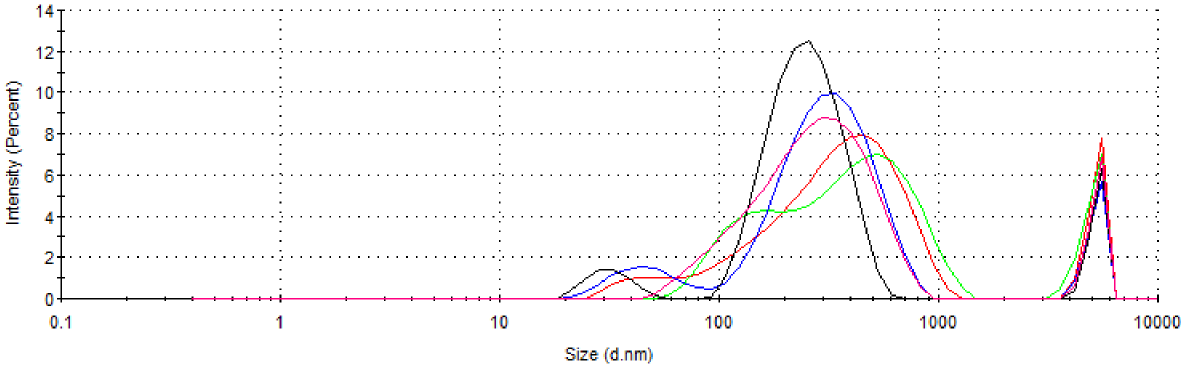

Table S2. **Aggregation of thiols, cross-linked polymers and model polymers**

Sample	V_H, mcl	C_H, M	V_G, mcl	C_G, M	PDI	$Z_{average}$, nm
**3**	150	10^{-3}	0	0	0.670±0.117	743±214
	150	10^{-4}	0	0	0.591±0.148	874±352
	150	10^{-5}	0	0	0.425±0.059	845±303
**3/4S**	1500	10^{-3}	0	0	0.435±0.091	375±35
	150	10^{-3}	0	0	0.476±0.133	467±61
	150	10^{-4}	0	0	0.375±0.065	674±167
**3/3S**	1500	10^{-3}	0	0	0.606±0.218	1383±1259
	150	10^{-3}	0	0	0.678±0.115	1810±422
	150	10^{-4}	0	0	0.650±0.174	782±254
**$(4S)_n$**	1500	10^{-3}	0	0	0.367±0.116	536±20
	150	10^{-3}	0	0		
	150	10^{-4}	0	0		
	150	10^{-5}	0	0		
**$(3S)_n$**	1500	10^{-3}	0	0	0.422±0.055	1583±139
	150	10^{-3}	0	0		
	150	10^{-4}	0	0		
	150	10^{-5}	0	0		
**Moxi**	0	0	15	10^{-2}	0.589±0.190	1504±441
	0	0	15	10^{-3}		
	0	0	15	10^{-4}		
**BCl**	0	0	15	10^{-2}	**0.236±0.058**	209±21
	0	0	15	10^{-3}		
	0	0	15	10^{-4}		
**3/4S: Moxi**	150	10^{-4}	15	10^{-2}	**0.295±0.035**	373±17
	150	10^{-5}	15	10^{-3}	**0.276±0.040**	446±13
	150	10^{-4}	15	10^{-4}	0.374±0.078	600±284
**3/3S: Moxi**	150	10^{-4}	15	10^{-2}	0.410±0.154	860±426
	150	10^{-5}	15	10^{-3}	0.337±0.048	617±203
	150	10^{-4}	15	10^{-4}	0.447±0.156	525±120
**$(4S)_n$: Moxi**	150	10^{-4}	15	10^{-2}	**0.235±0.025**	561±7
	150	10^{-5}	15	10^{-3}		
	150	10^{-6}	15	10^{-4}		
**$(3S)_n$: Moxi**	150	10^{-4}	15	10^{-2}	0.618±0.296	1786±114
	150	10^{-5}	15	10^{-3}		
	150	10^{-6}	15	10^{-4}		
**3/4S: BCl**	150	10^{-4}	15	10^{-2}	0.383±0.103	554±128
	150	10^{-5}	15	10^{-3}		
	150	10^{-6}	15	10^{-4}		
**3/3S: BCl**	150	10^{-4}	15	10^{-2}	**0.294±0.050**	556±97
	150	10^{-5}	15	10^{-3}		
	150	10^{-6}	15	10^{-4}		
**$(4S)_n$: BCl**	150	10^{-4}	15	10^{-2}	**0.269±0.031**	533±130

	150	10^{-5}	15	10^{-3}		
	150	10^{-6}	15	10^{-4}		
**$(3S)_n$: BCl**	150	10^{-4}	15	10^{-2}	0.351±0.192	1898±202
	150	10^{-5}	15	10^{-3}		
	150	10^{-6}	15	10^{-4}		

5. UV-vis study

Figure S25. Absorption spectra of macrocycle **3** (1×10^{-5} M) with a BCl solution (1×10^{-4} M) in the solvent system THF: CH_3OH = 100: 1

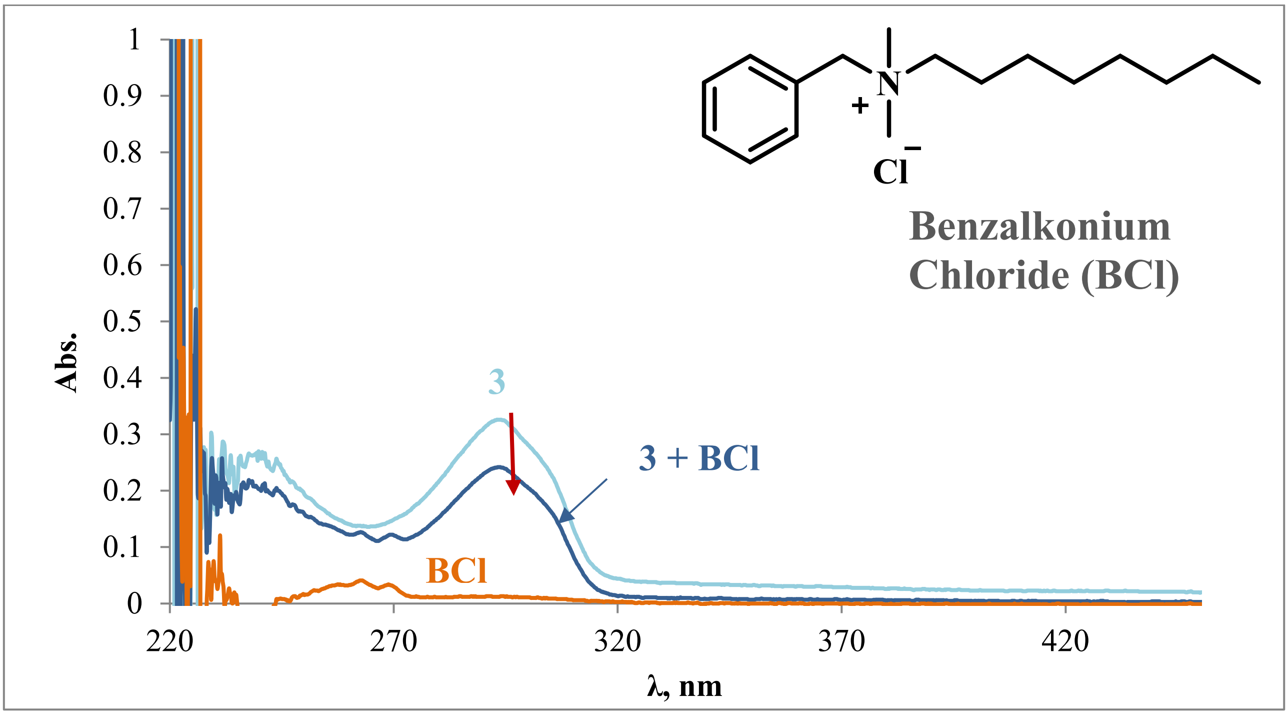

Figure S26. Absorption spectra of macrocycle **3** (1×10^{-5} M) with a **moxi** solution (1×10^{-4} M) in the solvent system THF: CH_3OH = 100: 1

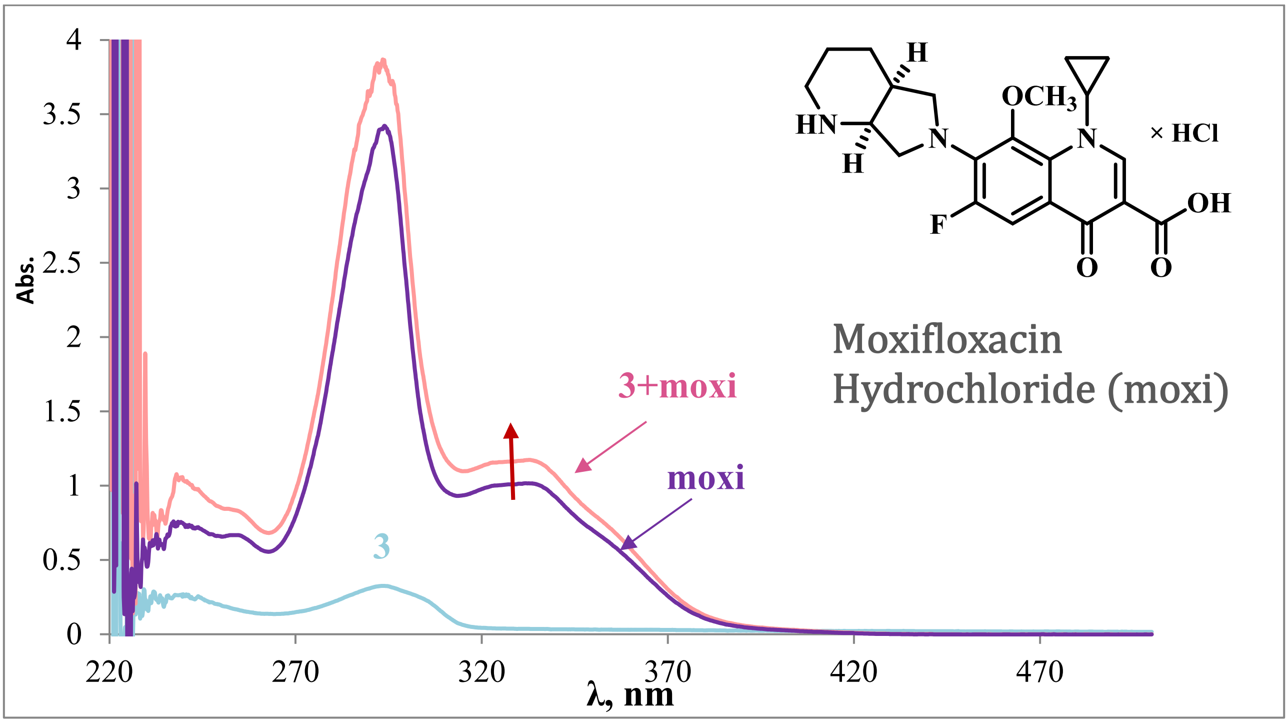

Figure S27. Titration curve for the system macrocycle **3** (0-2.67×10^{-5} M) / **moxi** (1×10^{-5} M) in solvent system THF: CH_3OH=100:1

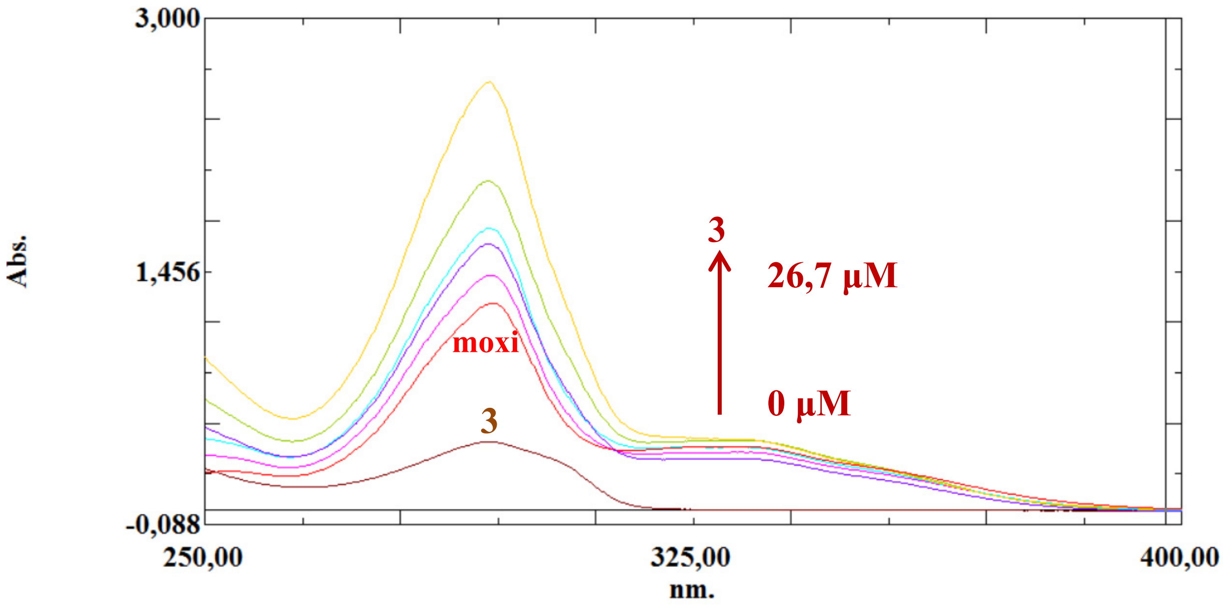

Figure S28. Bindfit (Fit data to 1:1, 1:2 and 2:1 Host-Guest equilibria) screenshots taken from the summary window of the website supramolecular.org. This screenshots shows the raw data for UV-vis titration of **3** with **moxi**, the data fitted to 1:1 binding model (a), 1:2 binding model (b) and 2:1 binding model (c).

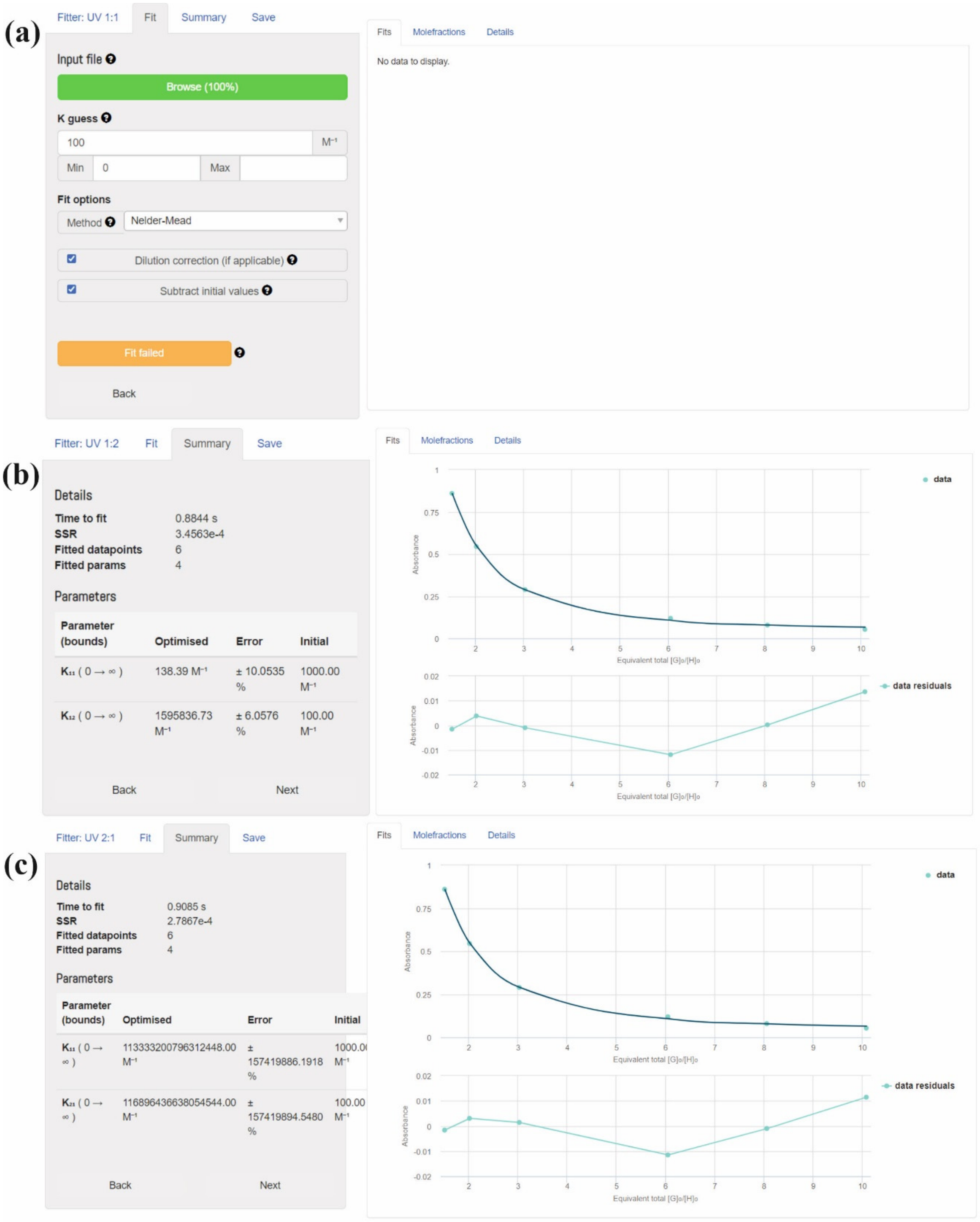

6. Chromatographic study of 3/3S, 3/4S, (3S)n, (4S)n.

Figure S29. GPC curves of products **3/3S** (eluent-THF, calibrated by PS standards)

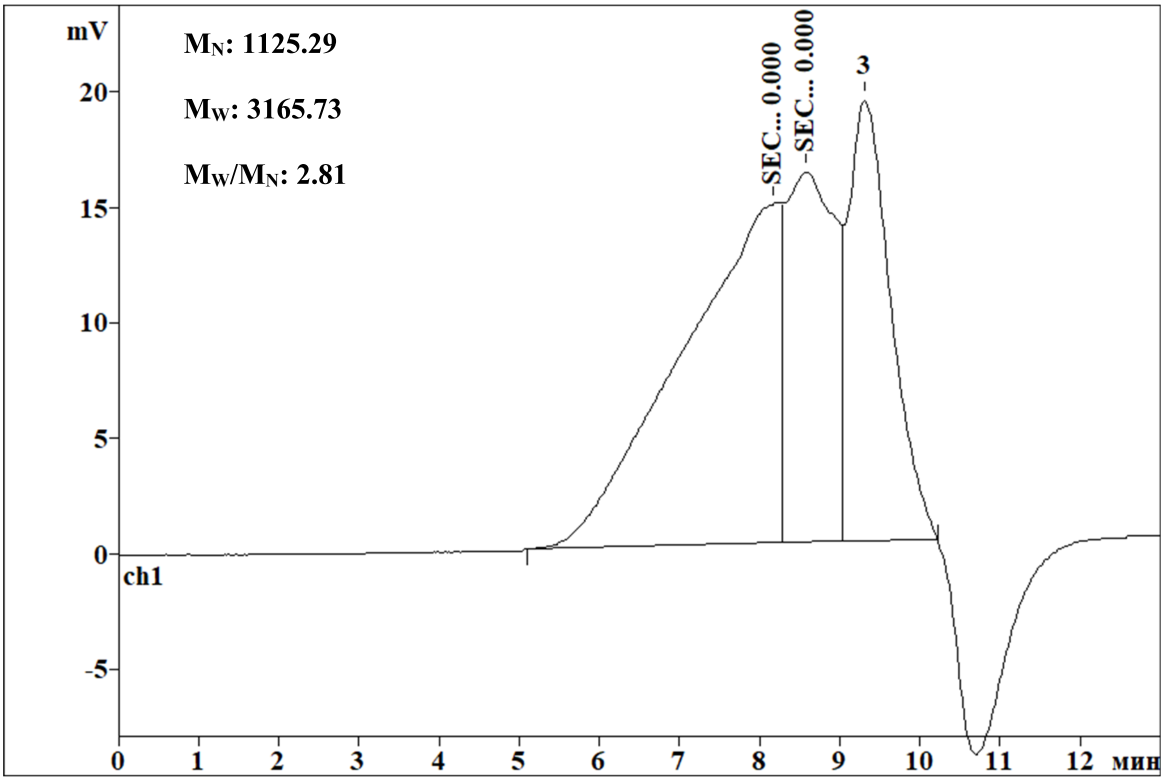

Figure S30. GPC curves of products **3/4S** (eluent-THF, calibrated by PS standards)

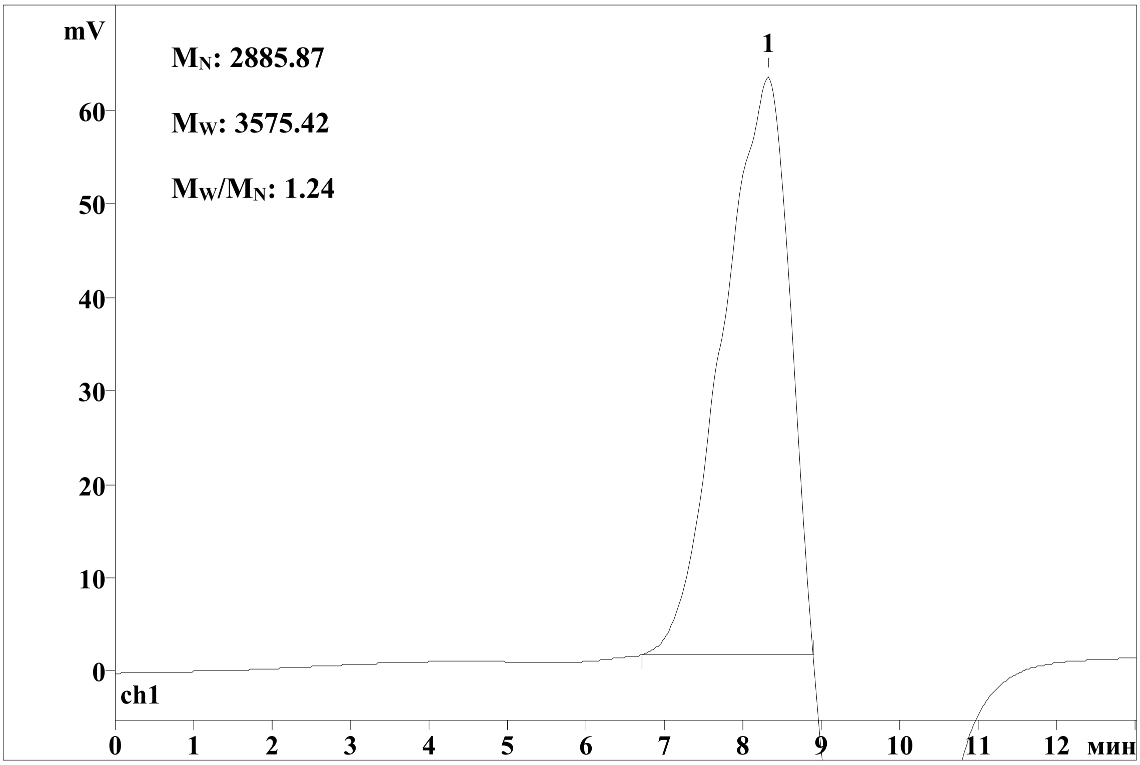

8. Thermal Gravimetric Analysis of 3, 3n, 3/3S, 3/4S

Figure S31. TGA (green) and differential scanning calorimetry (DSC) (blue) curves of **3**.

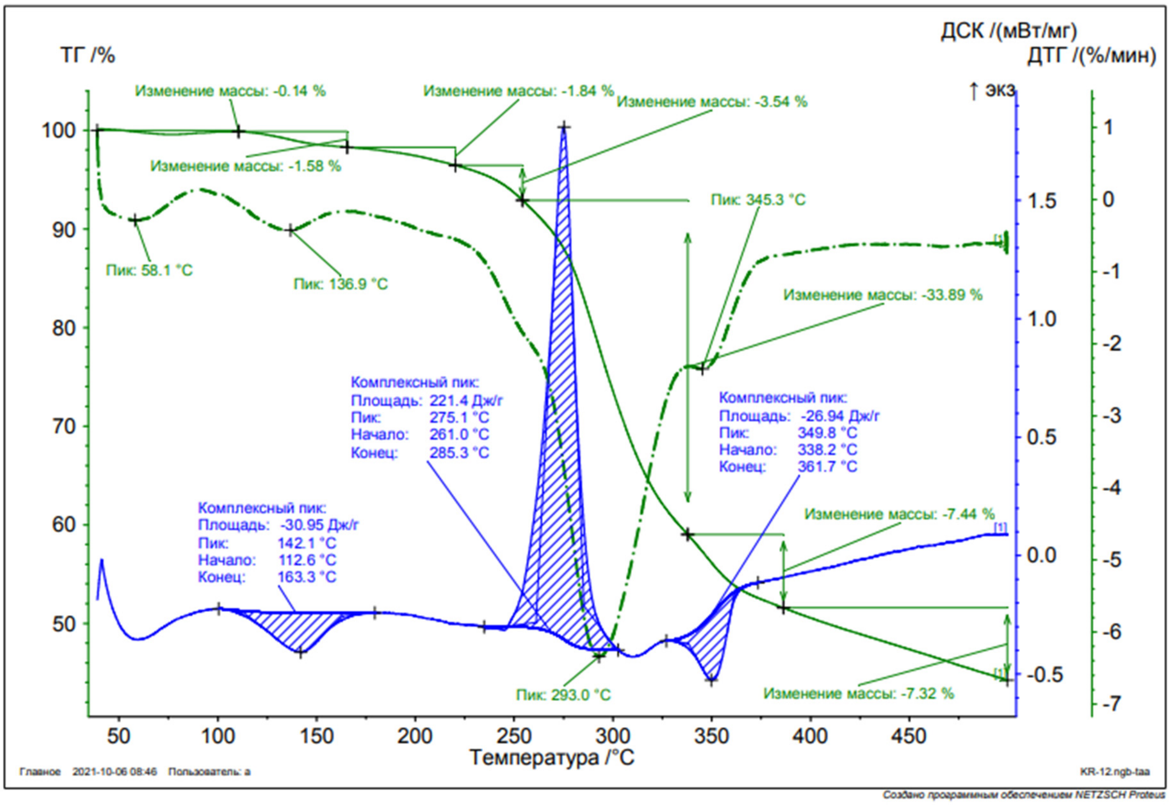

Figure S32. TGA (green) and differential scanning calorimetry (DSC) (blue) curves of **3n**.

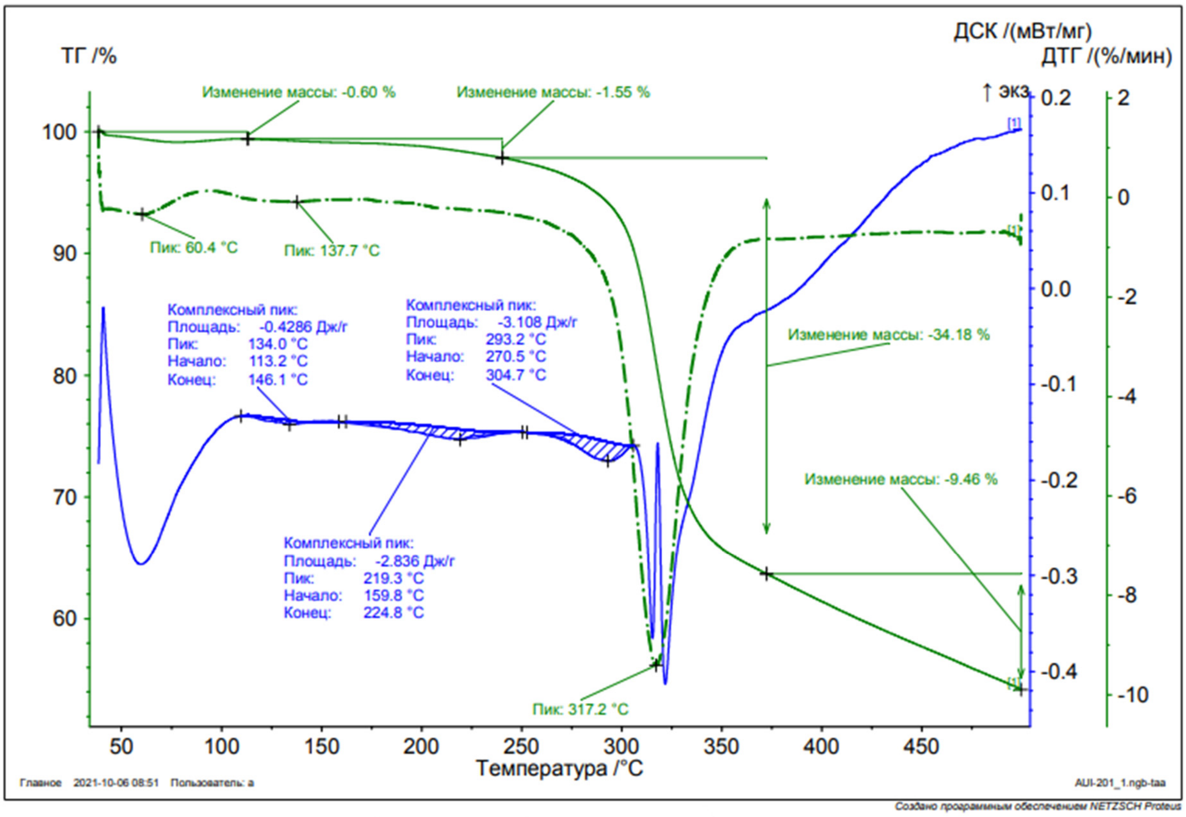

Figure S33. TGA (green) and differential scanning calorimetry (DSC) (blue) curves of **3/ 3Sn**.

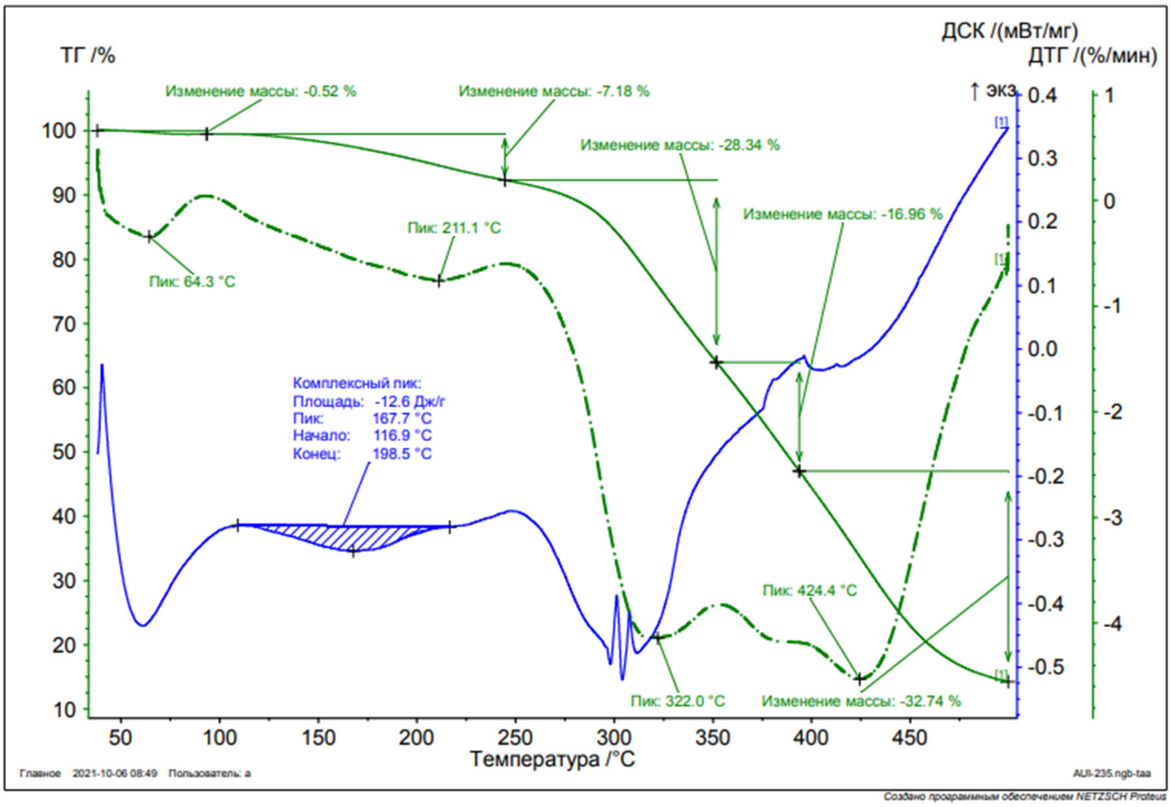

Figure S34. TGA (green) and differential scanning calorimetry (DSC) (blue) curves of **3/4Sn**.

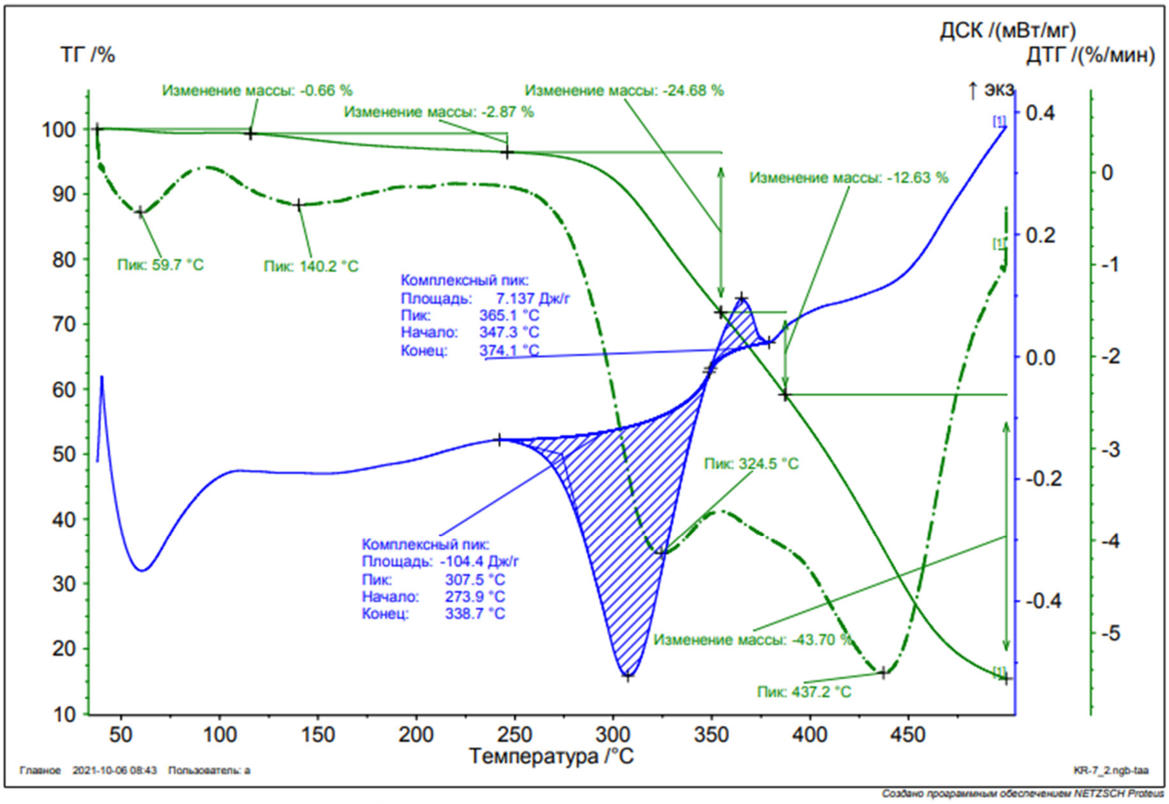

9. Morphology and Composition of 3/3Sn, 3/4Sn

9.1. Transmission Electron Microscopy

Figure S35. TEM image of **3/4S** (1×10^{-5}M) in the solvent system THF: CH_3OH (100:1) after the solvent evaporation.

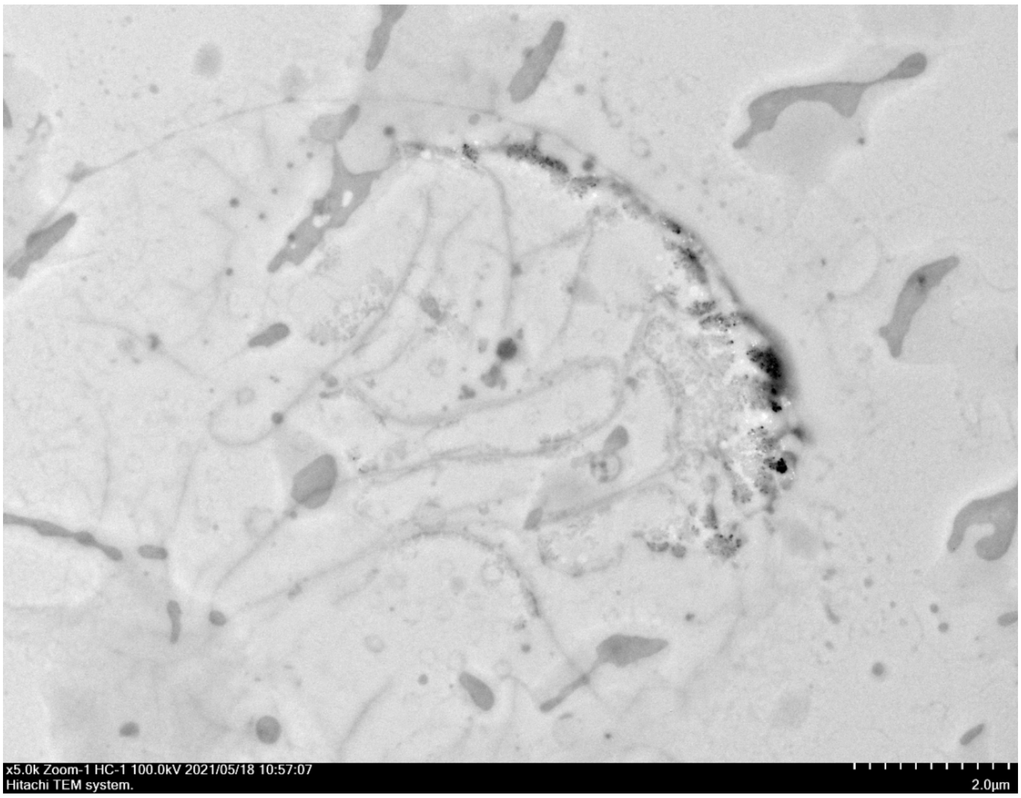

Figure S36. a-b) TEM image of **3/4S** (1×10^{-5}M) / **moxi** (1×10^{-4}M) in the solvent system THF: CH_3OH (100:1) after the solvent evaporation.

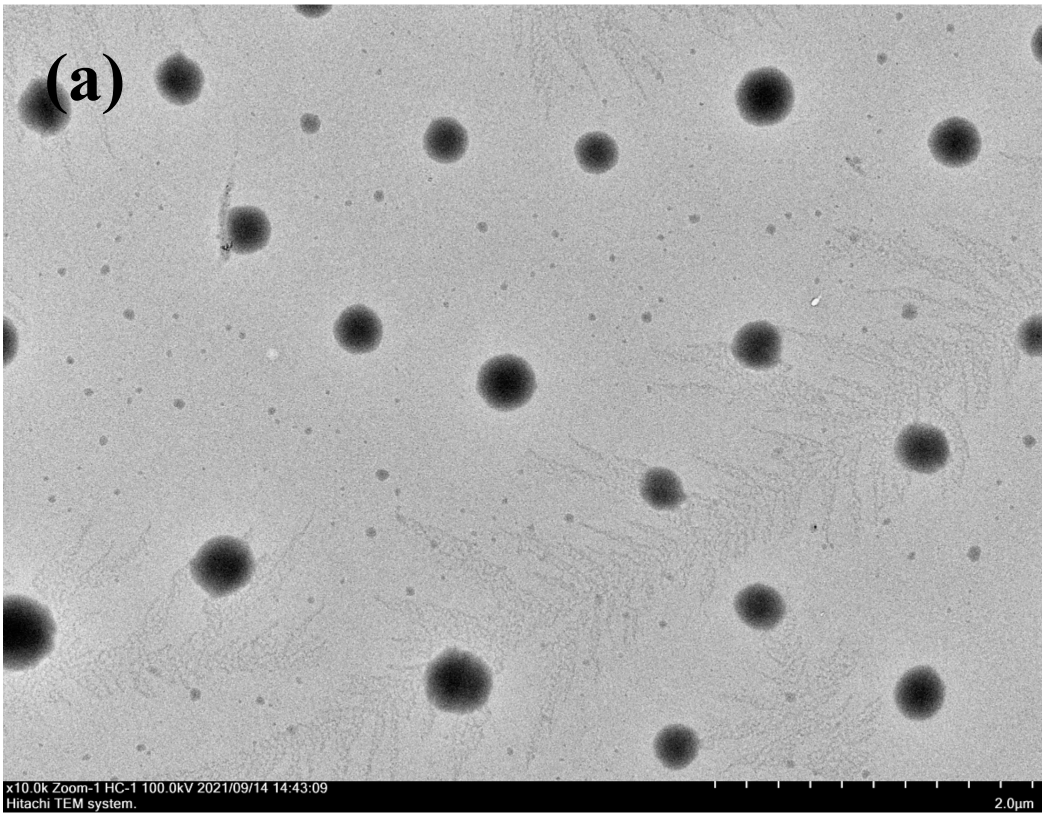

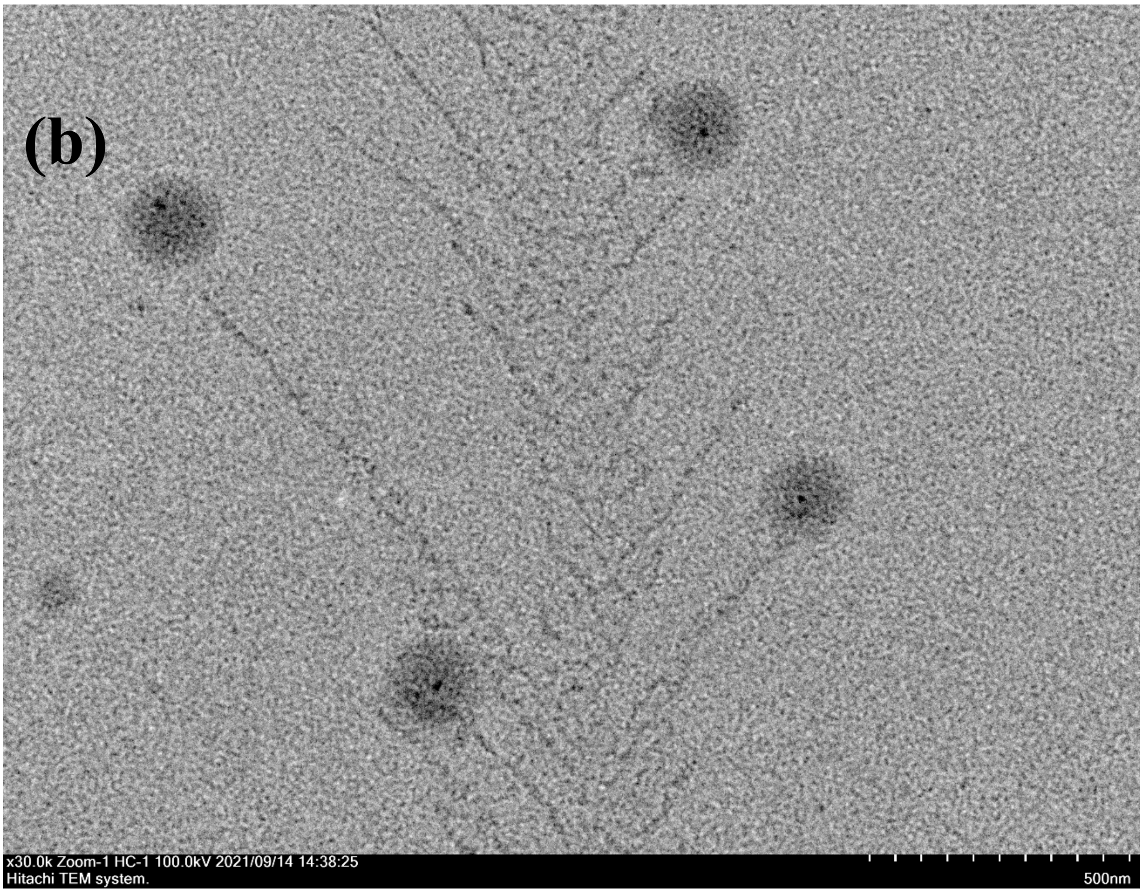

9.2. Scanning Electron Microscopy

Figure S37. a-b) SEM image of **3/4S** (1×10^{-5}M) in the solvent system THF: CH_3OH (100:1) after the solvent evaporation.

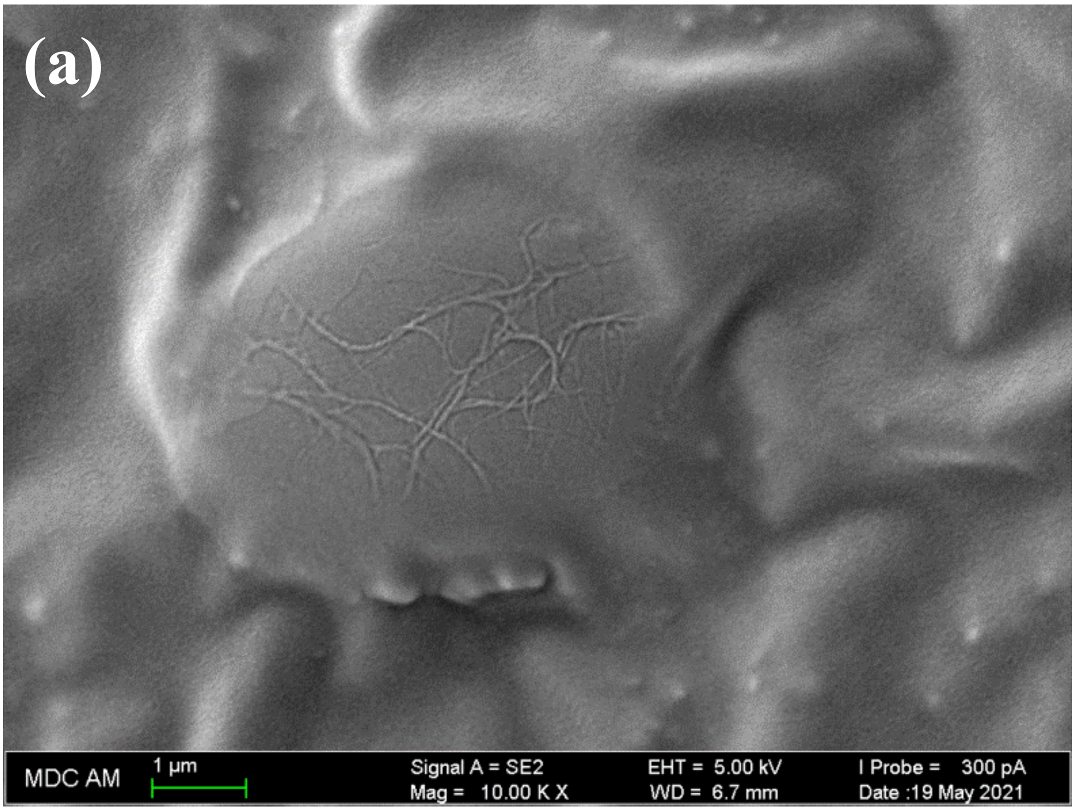

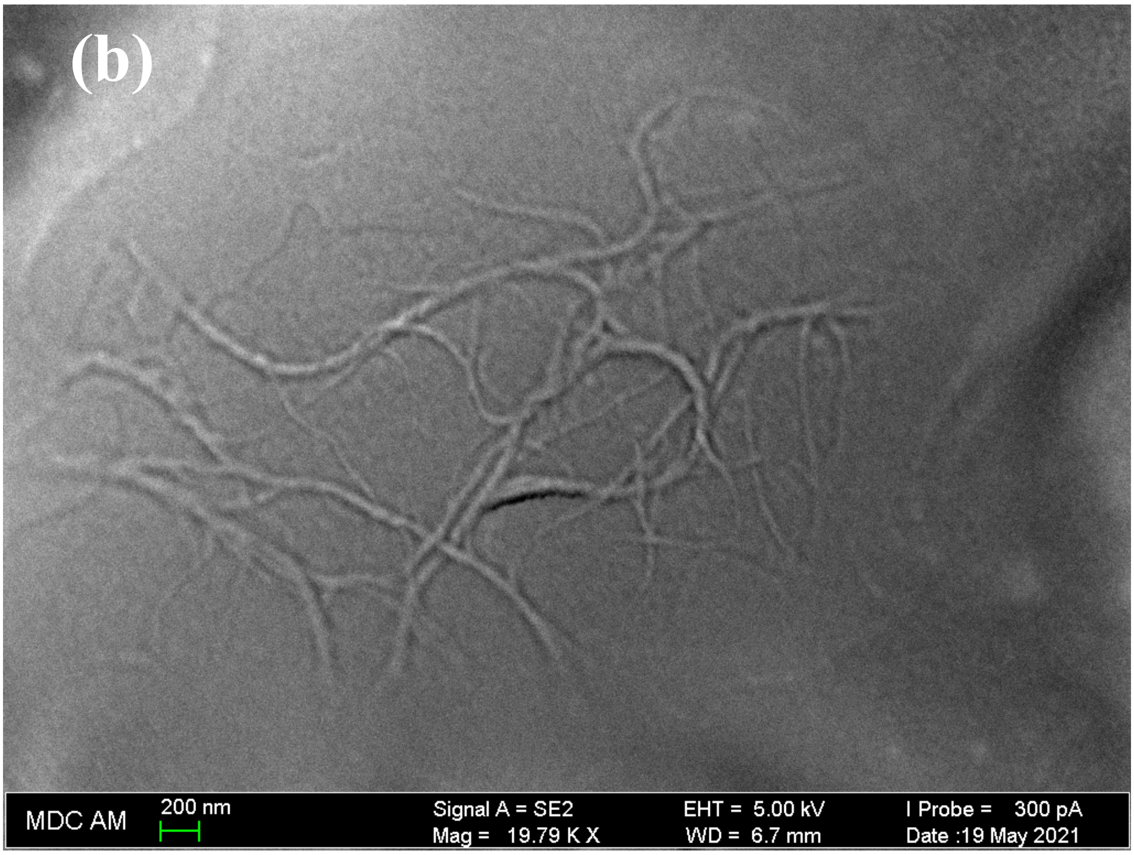

Figure S38. SEM image of **3/4S** (1×10^{-5}M) at low pressure in the solvent system THF: CH_3OH (100:1) after the solvent evaporation.

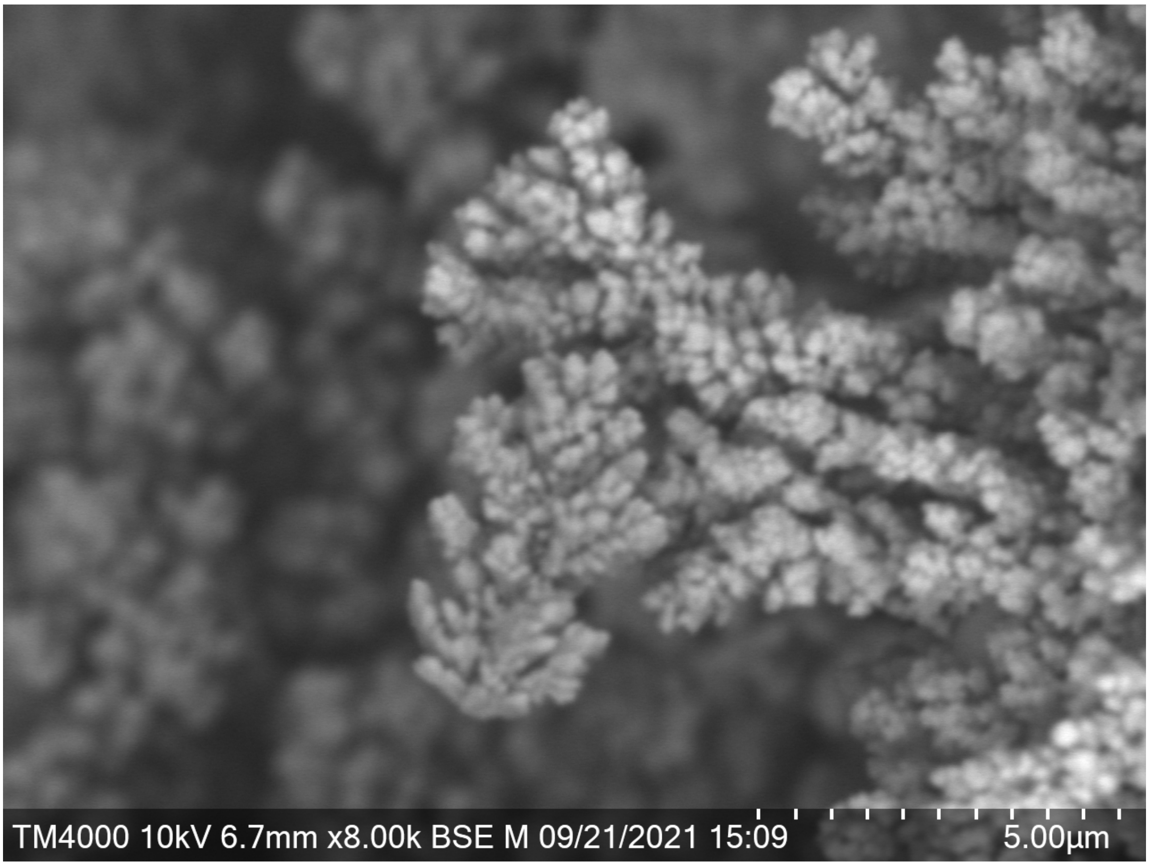

Figure S39. 3D-model of **3/4S** (1×10^{-5}M) film from SEM images at low pressure

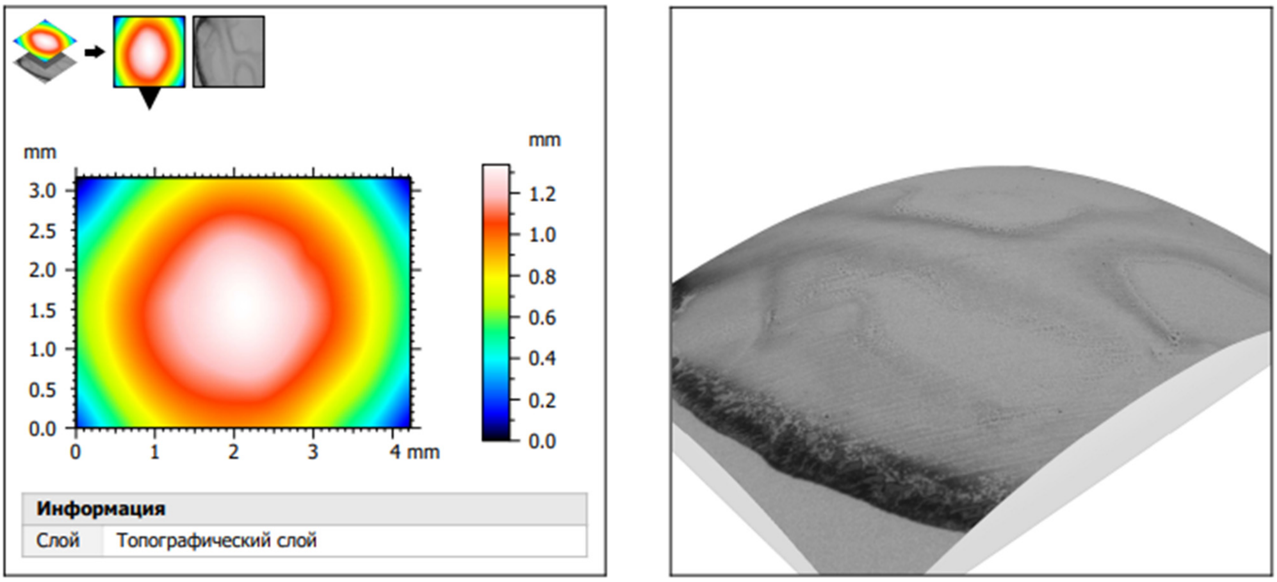

Figure S40. 3D-model of **3/4S** (1×10^{-5}M) / **moxi** (10^{-4}M) film from SEM images at low pressure

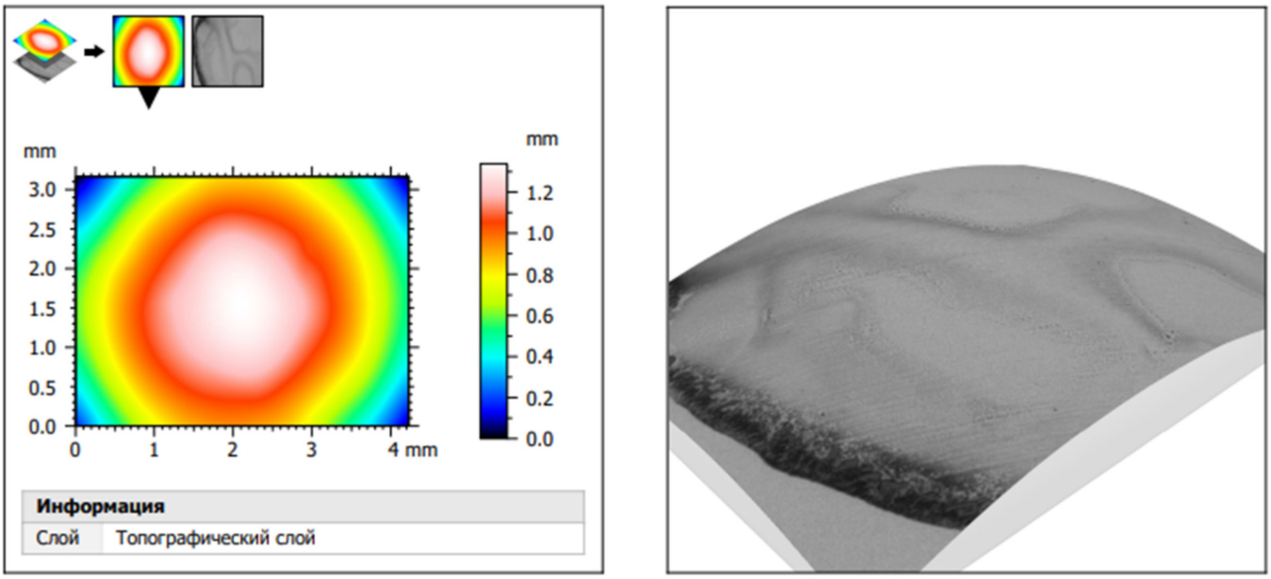

9.3 Atomic Force Microscopy

Figure S41. AFM image of **3/4S** (1×10^{-5}M) film in the solvent system THF: CH_3OH (100:1) after the solvent evaporation.

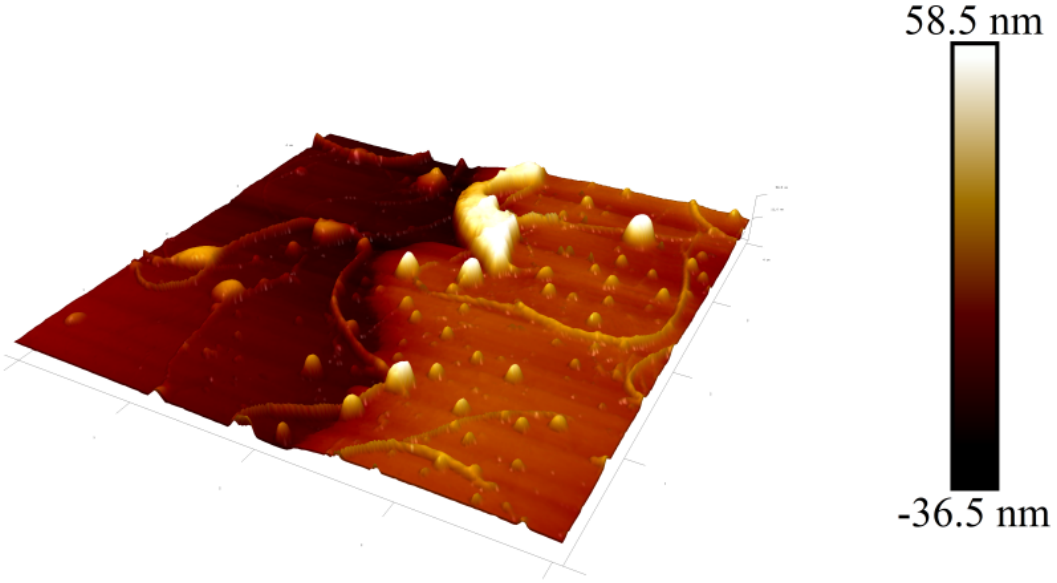

9.4 Optical Microscopy

Figure S42. Optical microscope of **3/4Sn** film in the solvent system THF: CH_3OH (100:1) after the solvent evaporation with surface disturbance.

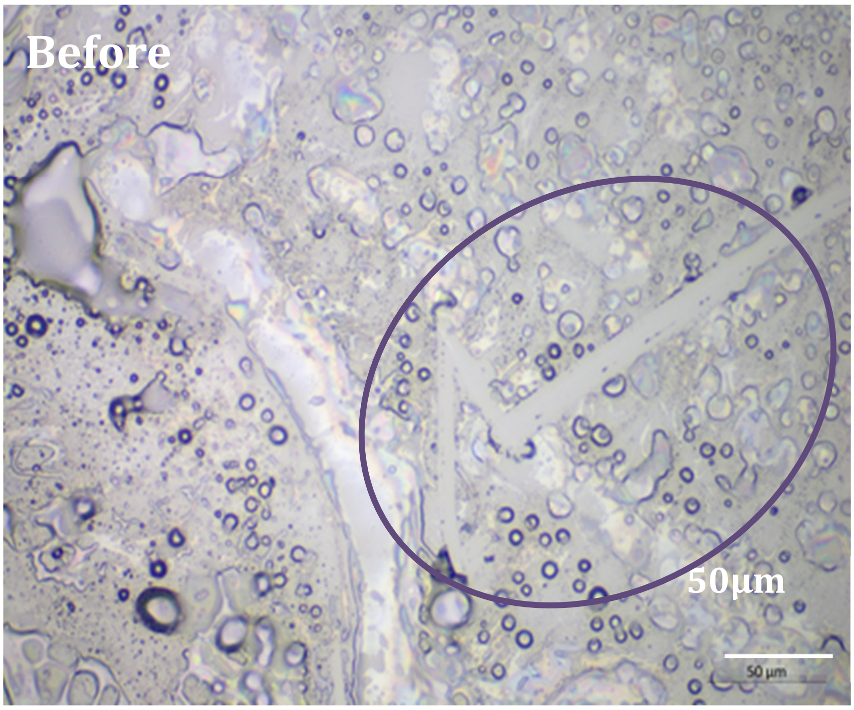

Figure S43. Optical microscope of **3/4Sn** film after 120 min during the H_2O_2 surface treatment after 120 minutes

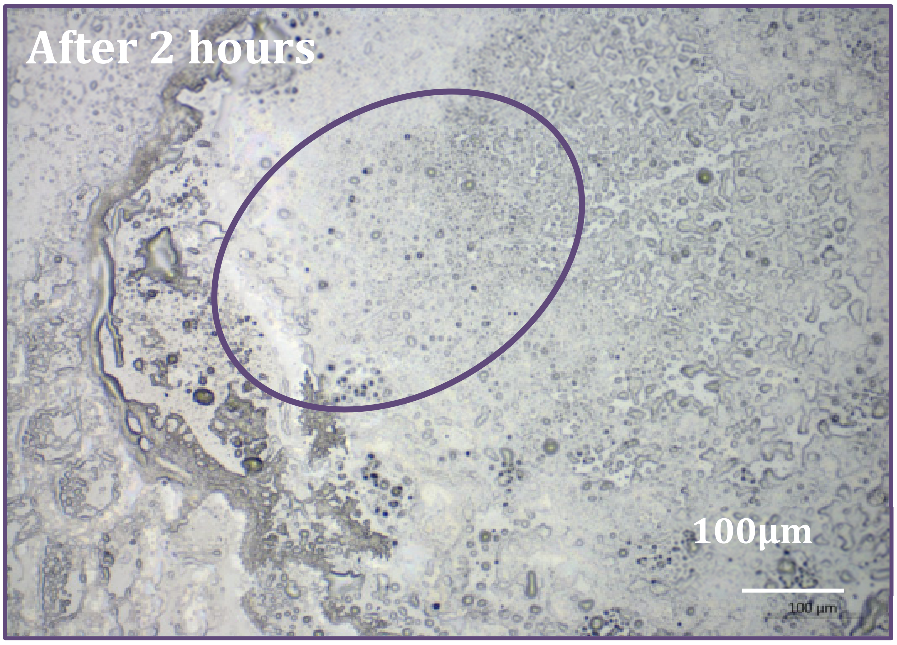

10. EPR study

Figure S44. EPR spectra of **3n** sample at room temperature in stationary (a) and in pulsed (b) modes of the X-band (9.6 GHz).

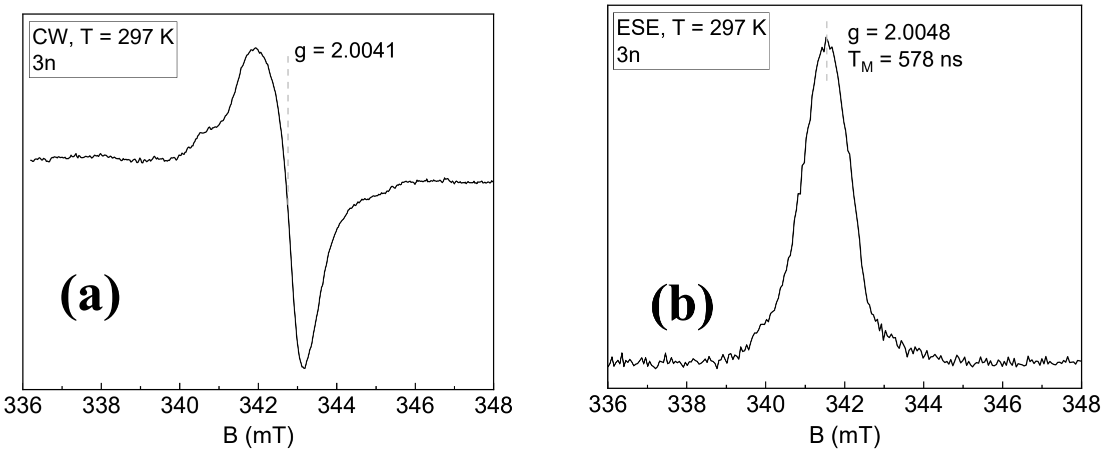

Figure S45. EPR spectra of **3/4Sn** film and **3/3Sn** film at room temperature before irradiation with an X-ray source.

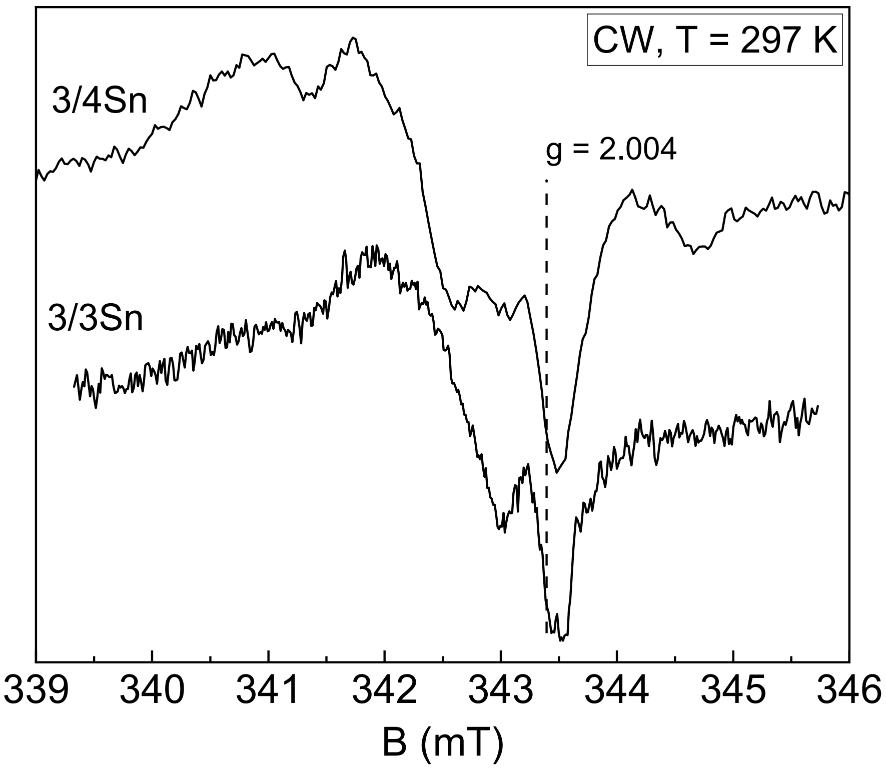

Supplement: Supplementary file 1 [file nanomaterials-12-01604-s001.zip › nanomaterials-1703127-supplementary.pdf]
